# Genomic Data Reveals Key Candidate Genes Underlying Chicken Comb Morphology

**DOI:** 10.1111/eva.70345

**Published:** 2026-10-24

**Authors:** Junjie Miao, Hao Chen, Guowen Song, Zhongwei Miao, Lili Zhang, Qingwu Xin, Li Li, Xiaopan Liu, Liang Huang, Qinlou Huang, Xueming Yan, Nenzhu Zheng, Zhiming Zhu

**Affiliations:** ^1^ Fujian Key Laboratory of Animal Genetics and Breeding, Institute of Animal Husbandry and Veterinary Medicine Fujian Academy of Agricultural Sciences Fuzhou China; ^2^ College of Life Sciences Jiangxi Science and Technology Normal University Nanchang PR China

**Keywords:** carnation phenotype, embryogenesis, genome‐wide association, *HSF5*, vascular development

## Abstract

Comb morphology is a salient chicken trait of biological and breeding interest. We analyzed whole‐genome data from 228 birds representing 10 breeds, including Hetian (HTC) and Xiangdong (XDC)—the only two Chinese breeds with carnation combs—to investigate population structure, diversity, and genomic correlates of comb type. Population analyses revealed clear breed stratification, with clustering that partly tracked comb type; breeds subjected to strong artificial selection or bottlenecks showed reduced genetic diversity. The integrated fixation index (*F*_ST_) and genome‐wide association study (GWAS) approach highlighted multiple regions differentiating carnation‐ from non‐carnation‐comb birds, with the strongest, most consistent signal at *HSF5*. Additional associated loci included *EDN3, ITGB8, PDE4B, SDK1, MC1R, CDKL5, NRXN1,* and *STAG3*. Gene‐set enrichment pointed to pathways involved in vascular development and embryogenesis, processes plausibly contributing to comb morphogenesis. Notably, while HTC and XDC maintained distinct global genomic backgrounds, they exhibited high similarity within the major candidate genes influencing the carnation comb phenotype, suggesting that independent breed differentiation occurred alongside a shared genetic basis for this trait. While causative variants were not established, these results nominate tractable candidates and pathways for functional validation and inform marker development for conservation and selection in indigenous breeds.

## 1 Introduction

The comb is among the most conspicuous phenotypic features of domestic chickens (*Gallus gallus domesticus*). Beyond its role as a classic secondary sexual trait, it provides a tractable model for studying morphological evolution and genetic regulation. Comb morphology conveys information on sex, age, health status, and social hierarchy; males with larger, well‐developed combs are often perceived as younger and healthier and consequently gain mating advantages (Parker 2003). The extensive variation in comb form (e.g., pea, rose, single) across natural populations and domesticated breeds (Hutt 2003) reflects underlying and still incompletely resolved genetic architectures.

Comb morphology is also linked to environmental adaptation and productivity. Rose‐combed breeds have been reported to show enhanced cold tolerance (Romanov et al. 2023), whereas large single combs may facilitate thermoregulation in hot climates (Adedeji et al. 2015). Production traits co‐vary with comb characteristics: comb morphology associates with body weight at 300 days (Wan et al. 2018), and hen comb traits correlate with egg number, egg weight, and survival (Zhou et al. 2025). As an avian skin appendage, the comb develops through coordinated epidermal–mesenchymal interactions, endocrine signaling, and environmentally responsive pathways (Mukhtar and Khan 2012), offering a useful system for probing gene–environment interplay in vertebrate organogenesis.

Recent genomic studies have identified major‐effect loci for specific comb phenotypes, illustrating diverse mutational mechanisms. Pea‐comb arises from dysregulated *SOX5* expression during development (Wright et al. 2009); rose comb is caused by a 7.4‐Mb inversion on chromosome 7 that repositions *MNR2* (Imsland et al. 2012); and duplex‐comb results from a ~20‐kb duplication upstream of *EOMES* (Dorshorst et al. 2015). Nevertheless, the broader regulatory network governing comb morphogenesis remains incomplete, and many morphological variants lack mechanistic explanation. Owing to its high vascularization, the comb has also been used as a model to investigate angiogenesis and immune regulation—particularly in contexts of necrosis or malformation (Chen et al. 2013)—and comb‐derived hyaluronic acid and its rapid scarless regeneration mechanisms offer novel insights for tissue regeneration research (Graciela et al. 2023).

Hetian Chicken (HTC), native to Changting County, Fujian Province, China, and Xiangdong Chicken (XDC), from Wuping County in the same province, share yellow plumage and integument yet uniquely exhibit the carnation‐comb phenotype—an upright single comb with a posterior bifurcation (Figure 4e) (Chen et al. 2011). Here, using whole‐genome data from 228 individuals representing nine Chinese indigenous breeds and one North American breed, we analyze genetic diversity and population structure and then contrast birds grouped by comb type. By integrating fixation index (*F*_ST_) scans with genome‐wide association study (GWAS) signals, we aim to identify candidate loci and pathways under positive selection during domestication and breeding, with particular attention to determinants of the carnation comb. These results refine current understanding of the genomic basis of comb morphology and provide a foundation for conservation and targeted improvement of carnation‐comb populations.

## 2 Methods

### 2.1 Experimental Animals

We analyzed 228 chickens from nine Chinese indigenous breeds and one North American breed, selected based on geographic distribution and phenotypic characteristics (Table 1). Whole blood was collected by subwing (brachial) venipuncture from 192 birds representing seven Chinese indigenous breeds: Hetian Chicken (HTC, *n* = 30), Bairong Chicken (BRC, *n* = 17), Dehua Chicken (DHC, *n* = 30), Jinhu Chicken (JHC, *n* = 29), Minqing Chicken (MQC, *n* = 30), Xiangdong Chicken (XDC, *n* = 26), and Zhangzhou Chicken (ZZC, *n* = 30). All sampling and experimental procedures were approved by the appropriate Institutional Ethics Review Board (approval no. 202402FJ015) and strictly followed the national guidelines for the care and use of laboratory animals. Publicly available whole‐genome sequencing data for Chantecler Chicken (CTC, *n* = 8, NCBI BioProject: PRJNA720223), Emei Black Chicken (EMB, *n* = 6, NCBI BioProject: PRJNA306389), and Tibetan Chicken (TBT, *n* = 22, NCBI BioProject: PRJNA306389) were obtained from the NCBI database and integrated into downstream comparative analyses.

**TABLE 1:** Geographic origin and comb morphology of 228 chickens from 10 breeds.

Breed	Code	Sample size	Origin	Comb type
Bairong Chicken	BRC	17	Putian, Fujian, China	Walnut
Chantecler Chicken	CTC	8	Canada	Cushion
Dehua Chicken	DHC	30	Dehua, Fujian, China	Single
Emei Black Chicken	EMB	6	Emei, Sichuan, China	Single
Hetian Chicken	HTC	30	Changting, Fujian, China	Carnation
Jinhu Chicken	JHC	29	Taining, Fujian, China	Rose
Minqing Chicken	MQC	30	Minqing, Fujian, China	Single
Tibetan Chicken	TBT	22	Tibet Plateau, China	Single
Xiangdong Chicken	XDC	26	Wuping, Fujian, China	Carnation
Zhangzhou Chicken	ZZC	30	Zhangzhou, Fujian, China	Pea
Total		228		

*Note:* Code, unique identifier for each breed used in analyses; Origin, sampling locality recorded at city/county and provincial level (country shown where applicable); Comb type, morphological category (single, rose, pea, cushion, walnut, carnation).

### 2.2 Whole‐Genome Resequencing

Genomic DNA was extracted from the whole blood of 192 chickens across seven Chinese indigenous breeds—Hetian Chicken (HTC, *n* = 30), Bairong Chicken (BRC, *n* = 17), Dehua Chicken (DHC, *n* = 30), Jinhu Chicken (JHC, *n* = 29), Minqing Chicken (MQC, *n* = 30), Xiangdong Chicken (XDC, *n* = 26), and Zhangzhou Chicken (ZZC, *n* = 30)—using a standard phenol–chloroform protocol. DNA quality and quantity were assessed by 1% agarose gel electrophoresis and by *A*260/280 ratios (acceptable range 1.8–2.0). Paired‐end libraries (150 bp reads) were prepared following standard procedures and sequenced on an Illumina NovaSeq 6000 platform (Novogene, Beijing, China). The mean sequencing depth per genome was approximately 12×.

### 2.3 Genome‐Wide Variant Calling and Annotation

Adapter sequences and low‐quality bases were removed with Trimmomatic v0.32 (Bolger et al. 2014) using *Q* < 30 as the trimming threshold. Cleaned reads were then aligned to the *Gallus gallus* reference genome (GenBank assembly accession: GCA_016699485.1) using Burrows‐Wheeler Aligner (BWA) v0.7.17 (Li and Durbin 2009) with default parameters. Alignments were converted to BAM, sorted with SAMtools v1.10 (Li et al. 2009), and processed using a Sentieon v201711.03 DNAseq pipeline (Kendig et al. 2019) in accordance with GATK Best Practices for SNP discovery. PCR duplicates were removed and base qualities were recalibrated with Sentieon (LocusCollector, Realigner). Final base quality score recalibration was run with GATK v4.0.12 (McKenna et al. 2010). Subsequent filtering was based on minor allele frequency (MAF > 0.05) and site call rate (> 90%), yielding a high‐confidence SNP set for downstream analyses. Subsequent filtering retained SNPs with a site call rate > 90% and a minor allele frequency (MAF) > 0.05. Given our dataset of 228 diploid chickens (456 autosomal chromosome copies), this 5% threshold requires a minor allele to be present in at least 23 copies. This stringency was applied to minimize genotyping artifacts common to rare variants at moderate sequencing depth (~12×). Furthermore, it ensures adequate statistical power for downstream *F*_ST_ selection scans and GWAS, preventing ultra‐rare alleles from inflating differentiation metrics through localized sampling effects or genetic drift. Functional annotation of variants was performed with SnpEff v5.0 (Cingolani et al. 2012), classifying SNPs by genomic context (intergenic, intronic, exonic, UTRs, upstream/downstream, and splice‐site categories) and predicted coding impact (synonymous, missense, stop‐gain, stop‐loss, etc.).

### 2.4 Population Structure and Genetic Diversity

Because sex chromosomes differ in inheritance and effective population size, we excluded Z and W variants and retained autosomal SNPs for downstream analyses (selection scans, phylogeny, and population structure) to avoid sex‐linked confounding. Autosomal SNPs were filtered in PLINK v1.9 (Chang et al. 2015) with ‐‐maf 0.05 ‐‐geno 0.05. Identity‐by‐state (IBS) matrices were computed in PLINK and used to construct a neighbor‐joining (NJ) tree in PHYLIP v3.57c neighbor (Felsenstein 2021); trees were visualized in iTOL (Letunic and Bork 2021). Principal component analysis (PCA) was performed with GCTA v1.93.2 (Yang et al. 2011) using ‐‐grm; the first six PCs were plotted in R.

Ancestry proportions were estimated with ADMIXTURE v1.3.0 (Alexander et al. 2009) for *K* = 1–11; results were summarized with cross‐validation to guide *K* selection. To limit linkage effects, we pruned SNPs with PLINK ‐‐indep‐pairwise 50 10 0.2 prior to ADMIXTURE and PCA. This *r* ^2^ threshold represents a conventional benchmark widely adopted in avian population genomics to mitigate long‐range LD‐driven biases while retaining sufficient polymorphic sites to capture fine‐scale genetic stratification (Yuan et al. 2022; Zhao et al. 2021).

We quantified genetic diversity using eight distinct metrics to evaluate the genomic background from both individual and population perspectives: common SNPs (CSNPs), runs of homozygosity (ROH), the ratio of homozygous fragments to observed heterozygosity (*F*_roh_/*H*_o_), the inbreeding coefficient (*F*), intra‐population genetic distance (DST), linkage disequilibrium (LD), the proportion of polymorphic sites (*P*_n_), and heterozygosity (*H*_p_). To assess the nucleotide diversity of each breed, CSNPs were determined by filtering autosomal SNPs for each individual breed using PLINK with parameters ‐‐maf 0.2 ‐‐geno 0.1. The *P*_n_ was estimated for each breed based on the raw, unfiltered variant dataset, while *H*_p_ was calculated across 10‐kb sliding windows. LD was quantified as the squared correlation coefficient (*r* ^2^) between pairs of SNPs using the PLINK options ‐r2 ‐ld‐window‐kb 1000 ‐ld‐window‐r2 0 to reflect historical effective population sizes and demographic dynamics. To evaluate the pedigree‐free inbreeding levels, *F* was computed via the plink ‐‐het command, which utilizes a method‐of‐moments estimator based on the deviation of observed versus expected homozygosity under Hardy–Weinberg equilibrium; a larger *F* value reflects more frequent inbreeding events within the population. To quantify the fine‐grained kinship proximity within each breed, DST was derived as the mean individual pairwise 1—IBS (Identity‐by‐State) distance matrix using the ‐‐distance square 1‐ibs option, where a smaller DST value indicates closer genetic relationships among individuals within that specific population. Lastly, ROH and *F*_roh_/*H*_o_ were called using the parameters ‐‐homozyg ‐‐homozyg‐snp 30 ‐‐homozyg‐kb 30 ‐‐homozyg‐gap 100 ‐‐homozyg‐window‐het 1 ‐‐homozyg‐window‐missing 5. These settings were chosen to optimize the sensitivity of detecting both short and long ROH segments while minimizing false positives from sequencing errors or missing genotypes. A higher abundance and longer length of ROH indicate lower genetic diversity within the population. To determine whether individual‐level diversity metrics (including *F*, DST, and ROH length) significantly differed among breeds, a one‐way analysis of variance (ANOVA) followed by Tukey's honestly significant difference (HSD) post hoc test was performed. Statistical significance was set at *p* < 0.05.

### 2.5 Detection of Selective Signatures

To identify selection signals associated with comb morphology, we contrasted carnation‐comb chikens (HTC, XDC) against non‐carnation‐comb breeds. Analyses were restricted to autosomal SNPs. Pairwise *F*_ST_ between groups was computed using PLINK v1.9. The *F*_ST_ dataset was filtered using ‐‐maf 0.01 and ‐‐geno 0.1. Estimates were summarized in non‐overlapping 10‐kb windows, and windows containing fewer than 10 SNPs were excluded. The top 1% of *F*_ST_ windows were considered candidate differentiated regions. In parallel, a genome‐wide association study (GWAS) was performed for the same case–control grouping with GEMMA (Zhou and Stephens 2012). The GWAS dataset was filtered for minor allele frequency ≥ 0.005, per‐SNP missingness < 0.05, and individual missingness < 0.02. To reduce the influence of linkage disequilibrium when estimating relatedness, SNPs were pruned using ‐‐indep‐pairwise 50 5 0.5, and a centered genomic relationship matrix was estimated using GEMMA (‐gk 2). Association testing was performed with a linear mixed model that incorporated the genomic relationship matrix as a random effect, thereby accounting for genome‐wide relatedness and ancestry‐related covariance among individuals. The binary phenotype was coded as 1 for HTC/XDC and 2 for the control group. Appropriate multiple‐testing correction was applied to define significant and suggestive association signals, and quantile‐quantile plots were examined to assess genomic inflation.

Variants supported by the GWAS were extended by 5 kb upstream and downstream and intersected with annotated genes. Genes overlapping the top 1% of *F*_ST_ windows and the GWAS‐supported regions were defined as consensus candidate regions. These regions were considered candidates for prioritization rather than validated causal loci. Genes within candidate regions were subjected to Gene Ontology (GO) enrichment using the Metascape platform (Zhou et al. 2019).

### 2.6 Haplotype Analysis and Functional Annotation

To characterize candidate regions, we integrated gene‐centric resources (e.g., GeneCards) and literature available in public repositories (e.g., NCBI). Nucleotide diversity (*π*) and windowed heterozygosity (*H*_p_) were calculated in 10‐kb windows with VCFtools v0.1.16 (Danecek et al. 2011) and PLINK. Haplotypes were phased with Beagle v5.1 (Browning et al. 2018) and visualized as heatmaps using the *pheatmap* R package v1.0.12 (Kolde and Kolde 2015). Regional differentiation was further assessed by absolute relative allele‐frequency differences (absRAFdif) between test populations. Predicted functional impacts of SNPs were obtained with SnpEff v5.0 under default parameters.

## 3 Results

### 3.1 Identification of Variants

We sequenced 228 individuals at an average depth of ~12× and aligned reads to the chicken reference genome (GCA_016699485.1). After quality control (minor allele frequency, MAF > 0.05; call rate > 0.90), we retained 10,456,792 variants for downstream analyses. Genome‐wide SNP density, computed in 1‐Mb windows, averaged one variant per 99 bp; Chromosome 24 showed the highest density, whereas Chromosome 37 showed the lowest (Figure 1a; Table S1). Functional annotation indicated that most SNPs fell within transcribed regions (38.87%) and introns (37.95%), followed by downstream (8.81%), upstream (8.69%), intergenic (2.89%), and exonic (1.31%) categories, with the remaining 1.48% located in essential regulatory and splicing regions (Figure 1b; Table S2). Within coding sequences, we identified 154,179 missense, 547,671 synonymous, 180 initiator‐codon, 333 start‐lost, 903 stop‐gained, and 98 stop‐lost variants (Table S3). These functional classes provide candidates for genotype–phenotype association and selection analyses relevant to comb morphology.

**FIGURE 1:**
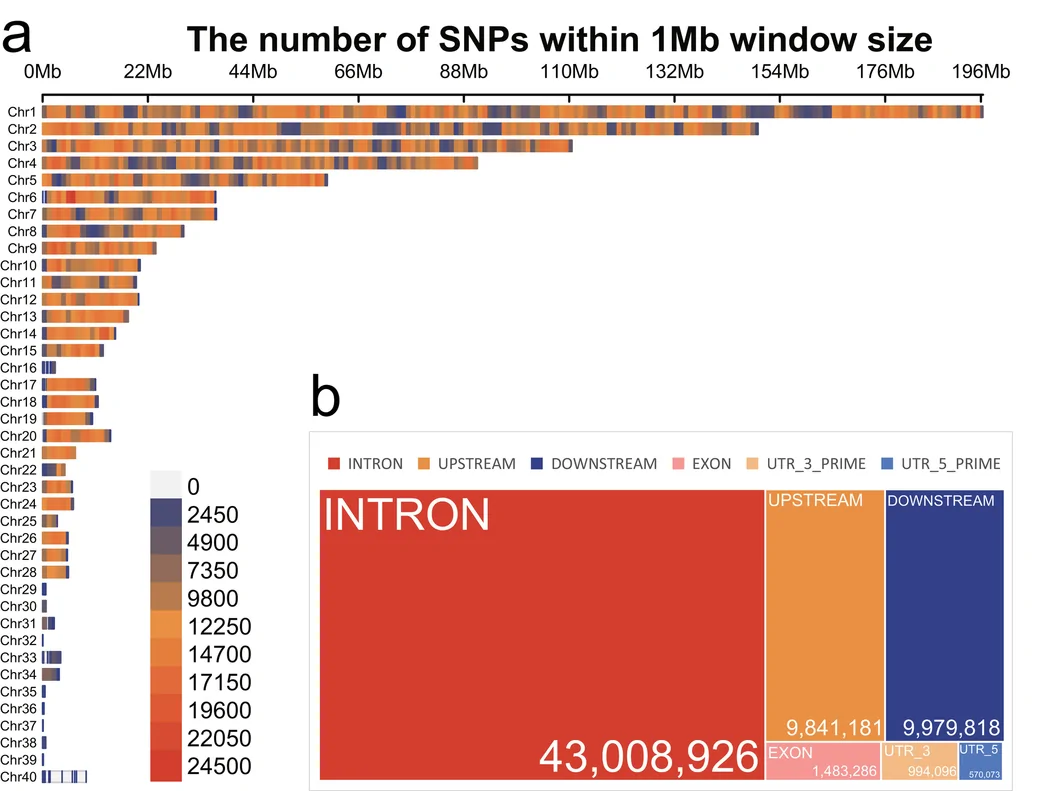
Genome‐wide SNP discovery and functional annotation in 228 chickens. (a) SNP density across the reference genome (GCA_016699485.1) calculated in non‐overlapping 1‐Mb windows (SNPs per Mb). (b) Functional distribution of annotated SNPs as reported by SnpEff (v5.0).

### 3.2 Population Structure and Genetic Diversity

Given the distinct inheritance patterns of sex chromosomes compared to autosomes, we excluded sex chromosomes and performed phylogenetic and population structure analyses using 10,158,173 autosomal SNPs. The neighbor‐joining tree grouped individuals by breed, supporting strong within‐breed genetic coherence (Figure 2a). PCA further resolved population structure: PC1 (3.22% variance) and PC2 (2.64%) separated most breeds into distinct clusters, and PC3 (2.50%) partitioned carnation‐comb from single‐comb groups (Figure 2b). LD decay differed among breeds: Tibetan Chicken (TBT) showed the fastest decay, whereas Chantecler (CTC) showed the slowest; Emei Black (EMB) and Bairong (BRC) also exhibited relatively slow decay (Figure 2c; Figure S1). ADMIXTURE analyses distinguished ancestries across a range of *K* values. At *K* = 6, carnation‐comb breeds (HTC, XDC) were clearly differentiated from other breeds; at *K* = 10, which was identified as the ideal solution with the lowest cross‐validation error (Figure S2), each of the 10 breeds constituted a distinct cluster, consistent with the NJ tree and PCA (Figure 2d).

**FIGURE 2:**
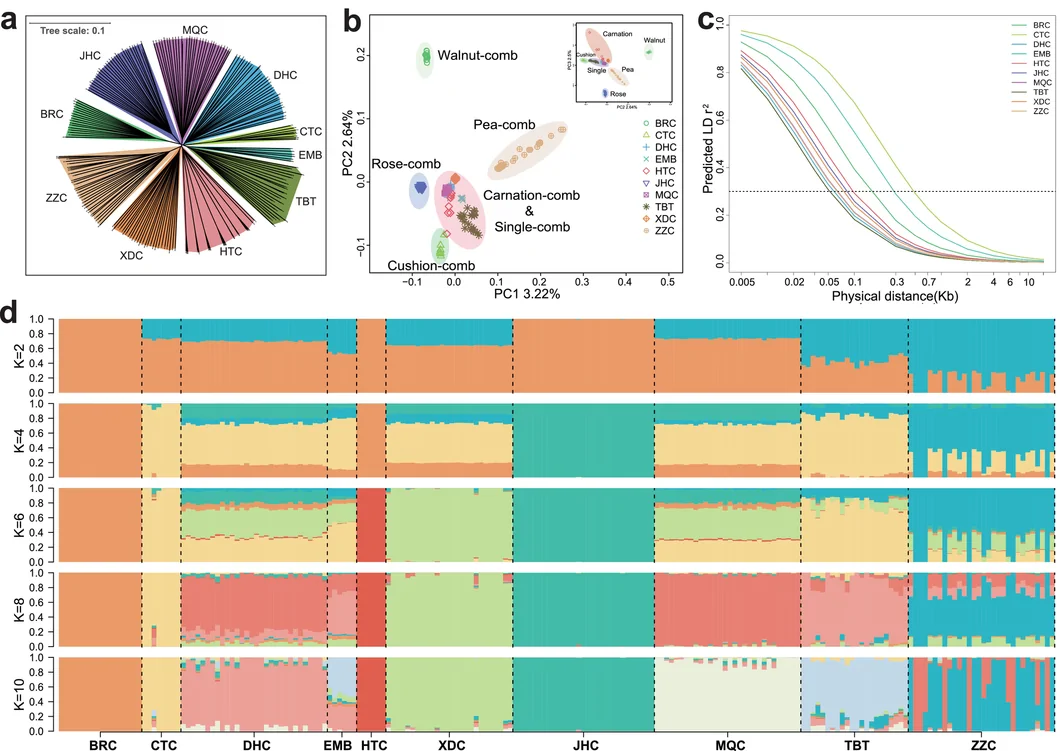
Population structure and diversity in 10 chicken breeds. (a) Neighbor‐joining (NJ) tree for 228 individuals constructed from identity‐by‐state distances using 10,158,173 autosomal SNPs. (b) Principal component analysis (PCA) of the same individuals, showing the first two (and third where indicated) principal components. (c) Linkage disequilibrium (LD) decay by breed, plotted as mean pairwise *r* ^2^ versus inter‐marker distance. (d) Ancestry proportions inferred by ADMIXTURE for *K* = 2–10 after LD pruning; bars represent individuals grouped by breed. Breed abbreviations are defined in Table 1.

We quantified eight diversity metrics across populations (Table 2; Table S4; Figure 3; Figure S3). The carnation‐comb (HTC, XDC) and single‐comb groups (DHC, EMB, MQC, TBT) generally displayed higher genetic diversity reserves, characterized by significantly higher CSNP counts and *P*_n_ values, balanced *H*_o_/*H*_e_ ratios, moderate DST, and consistently lower *F* and shorter ROH. By contrast, JHC exhibited markedly reduced heterozygosity (*H*_o_ = 0.24) and significantly extended ROH (6.45 Mb), whereas ZZC showed the same heterozygosity (*H*_o_ = 0.24) but an intermediate ROH length (3.31 Mb). In addition, BRC displayed the highest inbreeding coefficient (*F* = 0.33, *p* < 0.05) together with long ROH (11.28 Mb) and the lowest diversity indices (*H*_o_ = 0.20, *H*_e_ = 0.24, *P*_n_ = 0.75). Interestingly, while CTC had a lower *F* (*F* = 0.21), it exhibited the longest mean ROH (11.78 Mb) across all breeds alongside low expected heterozygosity (*H*_e_ = 0.21).

**TABLE 2:** Genetic diversity metrics for 10 chicken breeds grouped by comb morphology.

Breed	Comb	CSNP	*P*_n_	*H*_o_	*H*_e_	*F*	DST	ROH
BRC	Walnut	4,079,574	0.751	0.204	0.235	0.331a ± 0.045	0.211f ± 0.005	11.280a ± 3.436
CTC	Cushion	3,486,096	0.623	0.241	0.211	0.205bc ± 0.043	0.174 g ± 0.024	11.781a ± 2.947
JHC	Rose	4,460,678	0.888	0.242	0.266	0.215b ± 0.07	0.231d ± 0.008	6.454b ± 2.078
ZZC	Pea	4,687,069	0.920	0.242	0.273	0.184bc ± 0.068	0.239c ± 0.022	3.310 cd ± 6.604
DHC	Single	4,706,388	0.952	0.247	0.282	0.189bc ± 0.045	0.249ab ± 0.015	2.219 cd ± 1.618
EMB	Single	4,058,025	0.720	0.258	0.245	0.122bc ± 0.019	0.222e ± 0.018	0.724d ± 0.623
MQC	Single	4,766,909	0.948	0.258	0.282	0.156bc ± 0.046	0.244bc ± 0.012	2.776 cd ± 1.147
TBT	Single	4,616,781	0.935	0.252	0.283	0.121c ± 0.188	0.250a ± 0.020	2.330d ± 3.355
HTC	Carnation	4,734,325	0.890	0.264	0.275	0.120c ± 0.035	0.229de ± 0.069	1.101d ± 0.688
XDC	Carnation	4,531,779	0.905	0.246	0.273	0.186bc ± 0.048	0.238c ± 0.008	4.605bc ± 2.476

*Note:* Comb, visual classification based on external morphology; *P*_n_, proportion of polymorphic sites per breed; *H*_o_/*H*_e_, observed and expected heterozygosity; *F*, inbreeding coefficient computed from observed versus expected homozygosity; DST, average pairwise genetic distance (IBS‐derived); ROH length, summed length of runs of homozygosity per individual, in megabases; Breed abbreviations are defined in Table 1. For *F*, DST, and ROH length, values represent mean ± SD, and different superscript letters within the same column indicate statistically significant differences among breeds (*p* < 0.05, based on one‐way ANOVA followed by Tukey's HSD post hoc test; overall breed effects: *p* < 0.001).

Abbreviation: CSNP, common SNPs after quality control.

**FIGURE 3:**
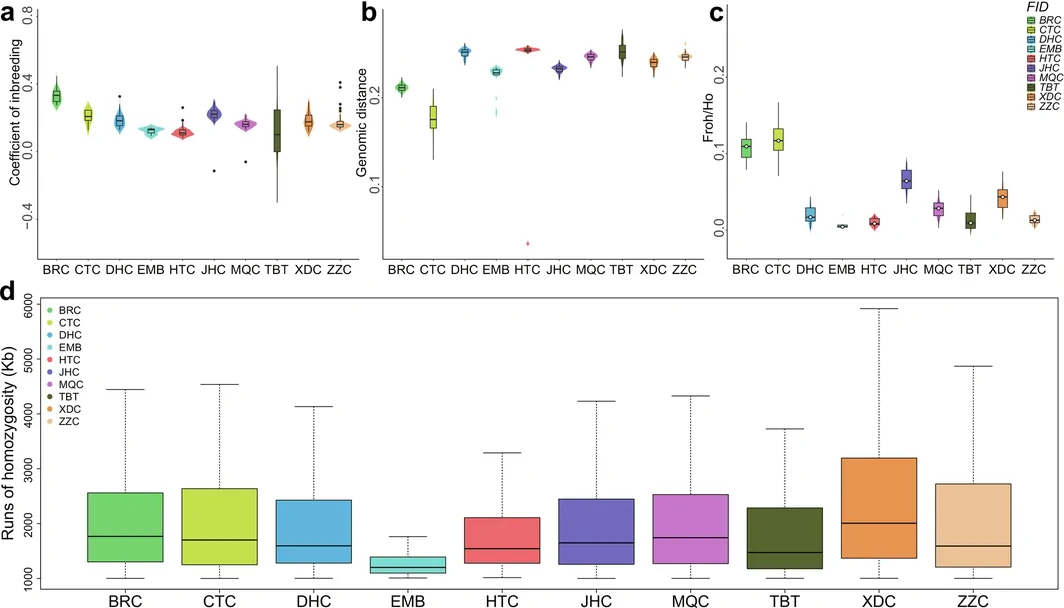
Genetic diversity across 10 chicken breeds. (a) Individual inbreeding coefficient (*F*) by breed. (b) Mean intra‐population genetic distance (DST) by breed. (c) Ratio of homozygous fragments to observed heterozygosity (*F*_roh_/*H*_o_) by breed. (d) Total length of runs of homozygosity (ROH) per individual (Mb), summarized by breed. FID in the figure key indicates breed; breed abbreviations are defined in Table 1.

### 3.3 Signatures of Selection Associated With Comb Type

To investigate the genetic basis of comb morphology, we contrasted carnation‐comb (HTC, XDC) versus non‐carnation‐comb breeds. Pairwise population differentiation identified 2035 candidate windows in the top 1% of the *F*_ST_ distribution (*F*_ST_ > 0.06), encompassing 348 annotated genes (Figure 4a; Table S5). The strongest *F*_ST_ peaks localized to *HSF5* on Chromosome 19 (*F*_ST_ = 0.23). Gene Ontology and KEGG enrichment implicated terms related to blood circulation, cell‐junction organization, cytoskeletal processes in muscle cells, and actin‐based functions (Figure 4b), consistent with putative roles in tissue development and circulatory physiology relevant to comb morphogenesis.

**FIGURE 4:**
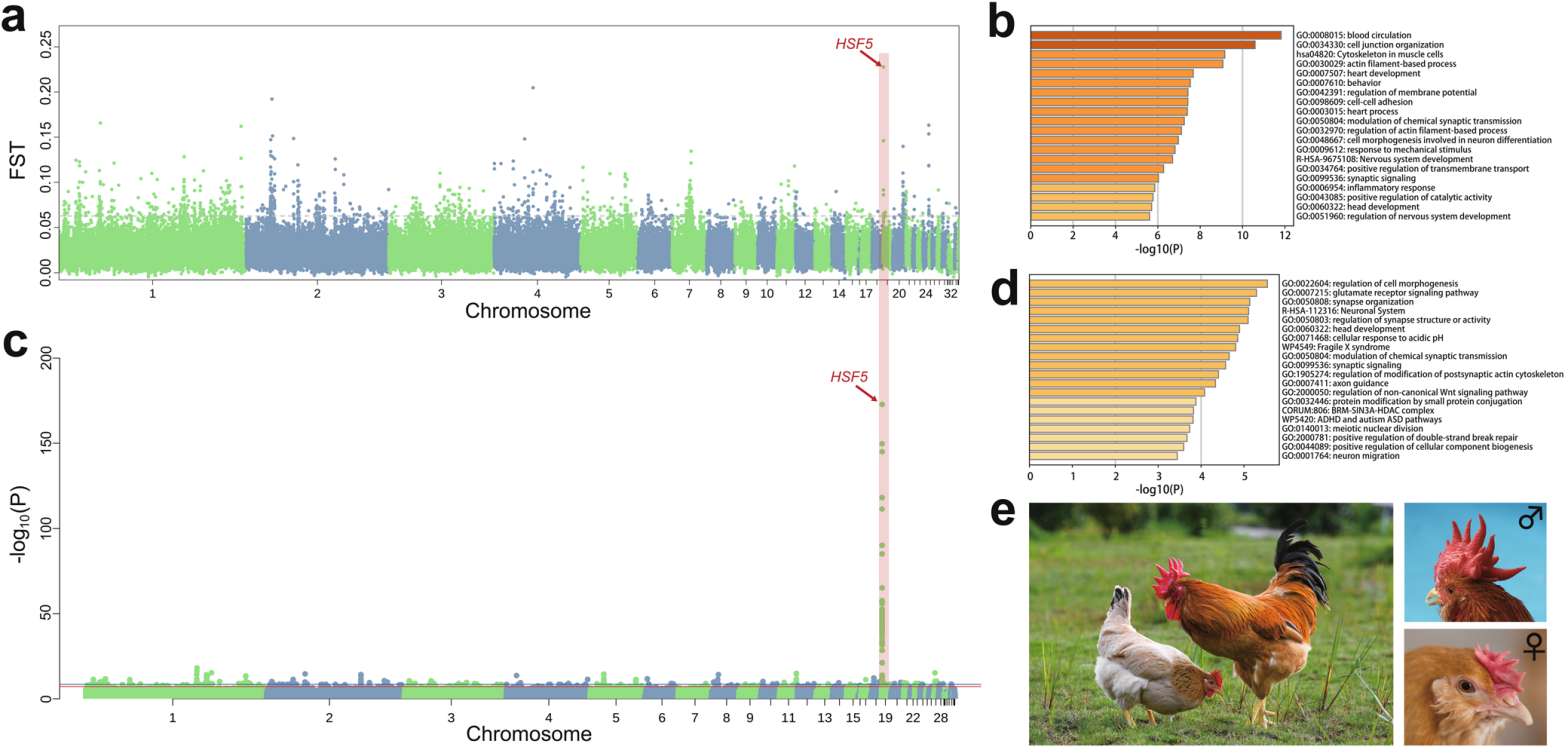
The carnation‐comb phenotype and associated selective signals. (a) Genome‐wide *F*_ST_ scan contrasting carnation‐comb (HTC, XDC) versus non‐carnation‐comb breeds (10‐kb windows; top 1% highlighted). (b) Significantly enriched Gene Ontology (GO) terms among genes in top *F*_ST_ windows. (c) GWAS contrasting the same groups; −log10(*p*) values shown genome‐wide (top 1% highlighted). (d) Significantly enriched GO terms among genes in top GWAS windows. (e) Representative photograph of the carnation comb, showing the characteristic three‐bladed morphology.

A complementary GWAS treating carnation comb as the case and non‐carnation comb as the control evaluated 14,889,349 SNPs (Figure 4c). The top 1% of associations (−log10(*p*) ≥ 7.17) comprised 2809 variants across 302 genes (Table S6), with the most significant peak again at *HSF5* on Chromosome 19 (−log10(*p*) = 172.79). Enrichment highlighted pathways involved in regulation of cell morphogenesis and glutamate receptor signaling (Figure 4d). Intersecting the top 1% *F*_ST_ and GWAS windows yielded 44 overlapping genes (including *HSF5*; Figure S4; Table S7), enriched for synapse assembly, regulation of filopodium assembly, and actin cytoskeleton organization—processes plausibly contributing to comb shape.

### 3.4 Candidate‐Gene Fine Mapping and Haplotypes

We focused on regions with strongest and most concordant signals between approaches. At *HSF5*, HTC and XDC exhibited haplotypes that were distinct from—and largely nonoverlapping with—those observed in the other breeds (Figure 5a). Consistent with a selective sweep, both carnation‐comb breeds showed reduced nucleotide diversity (π) and windowed heterozygosity (*H*_p_) at this locus (Figure 5b). Multiple *HSF5* variants exhibited large absolute relative allele‐frequency differences (absRAFdif) between carnation‐comb and other breeds (Figure 5c; Table S8). Three variants showed particularly high differentiation (absRAFdif > 0.63): an intronic SNP at position 1,130,544 (C>G), an upstream SNP at 1,134,274 (C>T), and a synonymous SNP in exon 2 at 1,130,314 (C>T). Although functional causality remains to be established, these variants are strong candidates for regulatory involvement at *HSF5* and provide tractable targets for follow‐up validation.

**FIGURE 5:**
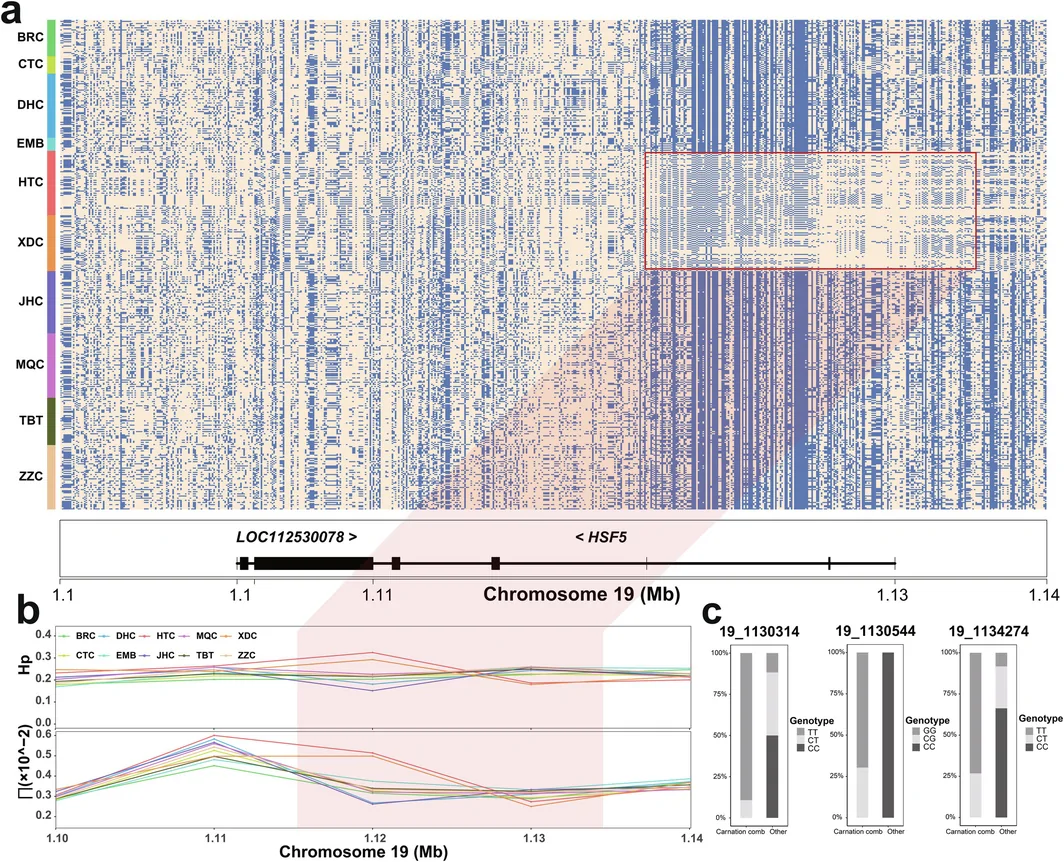
Differentiation at the *HSF5* locus between carnation‐comb and other breeds. (a) Haplotype configuration around *HSF5*; major and minor alleles are shown in beige and purple, respectively. Black rectangles indicate coding sequences (CDS). (b) Nucleotide diversity (π) and windowed heterozygosity (*H*_p_) in sliding windows spanning the *HSF5* region. (c) Genotype‐frequency distributions at three highly differentiated SNPs within *HSF5*.

The most pronounced phenotypic divergence between the two chicken groups lies in comb morphology. Focusing on genes potentially associated with the Carnation‐comb phenotype, we performed haplotype‐specific analyses by constructing heatmaps to resolve their haplotype configurations. We performed haplotype analyses on candidate genes, which revealed nearly fixed haplotypes in specific genomic regions, most notably at the *HSF5* locus (Figure 5a). Consistent with this, both HTC and XDC exhibited signatures of strong selective sweeps at this locus, as evidenced by reduced π and *H*_p_ values (Figure 5b). At the *HSF5* locus, we identified multiple SNPs with substantial absolute relative allele‐frequency differences (absRAFdif) between this carnation‐comb group and other breeds (Figure 5c; Table S8). Notably, three variants—an intronic variant (position 1,130,544; C>G), an upstream variant (1,134,274; C>T), and a synonymous variant in exon 2 (1,130,314; C>T)—showed pronounced allele frequency divergence (absRAFdif > 0.63) in both carnation‐comb breeds, suggesting potential regulatory impacts on *HSF5* expression.

## 4 Discussion

The comb is a prominent diagnostic trait in chickens, informative for breed identification, sexing, and health assessment, and a tractable model for studying morphological evolution and genetic regulation. While major recognized morphotypes (such as single, pea, rose, and walnut combs) have been well‐characterized (Figure S5), the carnation comb (Figure 4e) remains rare and comparatively understudied. Here, using two carnation‐comb breeds (HTC and XDC), we investigated the genomic architecture of comb morphology by analyzing whole‐genome data from 192 newly sequenced birds and 36 publicly available genomes, yielding 10,456,792 high‐quality SNPs across 228 individuals. Sex chromosomes and mitochondrial DNA have distinct inheritance patterns that can confound population analyses (Zhu et al. 2025). Specifically, in avian genomics, the heterogametic nature and distinct evolutionary dynamics of the Z and W sex chromosomes can introduce substantial heterogametic noise and sex‐specific stratification, primarily due to their smaller effective population size (*N*_e_) relative to autosomes (Feng et al. 2020; Mank et al. 2007). Retaining them can easily distort breed‐specific clustering patterns, particularly when sex ratios vary across sampled cohorts. Furthermore, from a biological standpoint, the core phenotype of interest—the carnation comb—is qualitatively and stably expressed in both male and female individuals within the Hetian Chicken and Xiangdong Chicken breeds. Because sexual dimorphism in poultry chiefly influences quantitative features such as comb size and vascular coloration rather than the fundamental qualitative morphology itself (Johnsson et al. 2012; Bakovic et al. 2022; Shen et al. 2016), focusing on autosomal variation effectively mitigates potential false‐positive selection signatures and genetic associations during downstream *F*_ST_ and GWAS that might otherwise be driven by sex‐linked stratification or uneven sex representation. Therefore, we restricted structure, diversity, and selection scans to 10,158,173 autosomal SNPs.

Tree‐, PCA‐, and ADMIXTURE‐based structure analyses jointly indicate pronounced partitioning by breed, with additional resolution of comb‐type groupings on higher PCs and at moderate‐to‐high *K*. This concordance across methods is consistent with discrete breeding histories and limited recent gene flow among the 10 populations. The LD profiles provide complementary information on within‐breed genetic backgrounds: the relatively fast decay in TBT is compatible with larger effective diversity, whereas the slower decay in CTC, EMB, and BRC is compatible with smaller effective diversity and/or stronger recent demographic or selective effects. Because LD is shaped by multiple forces (effective population size, recent drift, inbreeding, selection, and recombination landscape), these contrasts should be interpreted as indicative rather than causal.

Diversity metrics refine these inferences at the breed level. The carnation‐comb (HTC, XDC) and single‐comb (DHC, EMB, MQC, TBT) groups show comparatively elevated genetic variation (higher CSNPs and *P*_n_ with balanced *H*_o_/*H*_e_ and shorter ROH), which is consistent with relatively open breeding or reduced recent bottlenecks. In contrast, JHC and ZZC exhibit patterns compatible with stronger recent selection and partial diversity loss. The most severe signatures of genetic depletion were observed in BRC (walnut comb) and CTC (cushion comb), which present distinct demographic scenarios. BRC combined an elevated inbreeding coefficient with extended ROH, suggesting a regime of sustained, long‐term inbreeding. Conversely, CTC exhibited a lower *F* yet harbors the longest cumulative ROH, a classic genomic signature reflecting a severe recent bottleneck or intense recent artificial selection prioritizing the cushion‐comb phenotype. These patterns are highly compatible with historical population contractions and subsequent recovery from limited founders (Zhang et al. 2018; Darrington et al. 2023). Together, these results highlight heterogeneity in breeding histories across morphotypes and emphasize the value of targeted diversity management for rare‐breed conservation.

Contrasting carnation‐comb versus non‐carnation groups revealed discrete genomic signals associated with comb morphology. The intersection of *F*_ST_ and GWAS signals (44 genes) included pigmentation (*MC1R*; Guida et al. 2022), neurodevelopmental regulators (*CDKL5, NRXN1*; Leonard et al. 2022; Hu et al. 2019), a meiosis‐related factor (*STAG3*; Llano et al. 2014), and genes implicated in vascular development or signaling (*EDN3, ITGB8, PDE4B, SDK1*; Makita et al. 2008; Ma et al. 2016; Chen et al. 2024; Rochon et al. 2021). The prominence of vascular‐ and morphogenesis‐related terms is consistent with the comb's anatomy and development and suggests candidate routes by which genotype could modulate comb form.

Among these regions, *HSF5* on chromosome 19 showed the strongest and most concordant signal across methods. Although *HSF5* has not been functionally linked to comb development, recent work indicates roles in male fertility and meiosis with testis‐enriched expression (Liu et al. 2024; Yoshimura et al. 2024). Genes with testis‐biased expression frequently participate in early developmental programs (Chen et al. 2025), and carnation‐comb determination occurs during embryogenesis. In this context, *HSF5* is a plausible regulatory candidate whose effects need not be restricted to gonadal tissue. Consistent with selection at this locus, carnation‐comb breeds exhibited reduced π and *H*_p_ around *HSF5*, near‐fixed haplotypes distinct from other breeds, and multiple highly differentiated variants spanning intronic, upstream, and synonymous sites (absRAFdif > 0.63). These observations nominate *HSF5* for functional follow‐up but do not establish causality; alternative explanations (e.g., linkage to the causal variant, local recombination features) remain viable. However, our study is correlative; functional validation (e.g., expression profiling during embryonic comb development, reporter assays for regulatory variants, genome editing) will be required to test mechanistic hypotheses.

HTC and XDC are currently the only recognized Chinese chicken breeds with the carnation comb, and the case group therefore represents the complete range of recognized Chinese carnation‐comb breeds presently available. Although the signals are necessarily defined by this particular breed combination and may change under different sampling designs, several measures were taken to reduce confounding. Genome‐wide neighbor‐joining, PCA, and ADMIXTURE analyses identified HTC and XDC as genetically distinct breeds. Although shared local ancestry at a specific candidate region cannot be formally excluded using genome‐wide differentiation alone, this clear separation substantially reduces the concern that the observed signals simply reflect a single undifferentiated ancestral background. Moreover, local‐ancestry inference requires clearly defined reference populations (Maples et al. 2013), which are often unavailable for indigenous chicken breeds because of extensive historical admixture and the absence of discrete ancestral gene pools (Guo et al. 2022; Toalombo Vargas et al. 2019). We therefore did not treat regional haplotype similarity as independent evidence of phenotype association.

The control group was designed to minimize, as far as possible, confounding from traits other than comb morphology by including the Fujian breeds BRC, DHC, JHC, MQC, and ZZC, which differ in comb type and encompass variation in plumage color (black, white, and yellow), feathered and clean legs, body weight, and egg‐production performance, together with additional breeds from other geographic regions. This design reduces reliance on any single comparator and decreases the likelihood that the signals are driven by one breed‐specific trait. In addition, *F*_ST_ and GWAS signals were required to overlap, prioritizing regions supported by different ranking criteria. Although both analyses use the same case–control grouping and therefore cannot provide ancestry‐independent validation, their convergence provides method‐robust evidence and reduces the risk that the prioritized regions are artifacts of a single analytical approach. Together, these measures support the identified regions as context‐dependent but robust candidate signals within the present breed panel.

In conclusion, our study shows that Carnation‐comb chickens exhibit distinctive genomic patterns consistent with long‐term natural and artificial selection. Using whole‐genome data from 228 birds, we show that the two Chinese carnation‐comb breeds (HTC and XDC) are genetically distinct and differ in population structure from other comb‐type breeds. Integrating *F*_ST_ scans with GWAS highlighted genomic regions enriched for allelic differentiation in carnation‐comb populations. Concordant genomic signatures across both breeds prioritized a focused set of candidates—most notably *HSF5*, which showed the strongest and most concordant association across methods—together with genes and pathways implicated in vascular development and morphogenesis. These results nominate biologically plausible mechanisms for comb shape diversification and provide a ranked target list for functional validation. Practically, the findings offer informative markers for conservation management and trait‐focused selection in indigenous breeds, while establishing a genomic framework for dissecting additional comb morphotypes.

## Funding

This work was supported by National Key Research and Development Program of China (2024YFD1300902), Major Science and Technology Projects of Fujian Province (2024NZ029028), Fujian Academy of Agricultural Science Research Project (XTCXGC2021008).

## Ethics Statement

All experimental procedures were approved by the Institute of Animal Husbandry and Veterinary Medicine, Fujian Academy of Agricultural Sciences, Institutional Ethics Review Board (approval no. 202402FJ015) and strictly followed China's Guidelines for the Ethical Review of Animal Welfare in Laboratory Animals.

## Conflicts of Interest

The authors declare no conflicts of interest.

## Supporting information


**Table S1:** Variant counts and densities by chromosome in 228 chickens aligned to the GCA_016699485.1 reference.
**Table S2:** Genomic distribution of sequence‐variant annotations across functional categories in chickens.
**Table S3:** Functional classification and frequency distribution of variant types in the chicken cohort (*n* = 228).
**Table S4:** Comparison of population genetic parameters and ANOVA results among 10 breeds.
**Table S5:** Genes overlapping genomic windows in the top 1% of the *F*_ST_ distribution for carnation‐comb versus non‐carnation‐comb chickens.
**Table S6:** Genes overlapping genomic windows in the top 1% of GWAS signals for carnation‐comb versus non‐carnation‐comb chickens.
**Table S7:** Forty‐four candidate genes supported by overlapping *F*_ST_ and GWAS signals, with associated Gene Ontology (GO) enrichment terms.
**Table S8:** eva70345‐sup‐0001‐Supinfo.docx. *HSF5* absolute relative allele frequency(> 0.6)in carnation comb chicken against non‐carnation comb chicken.
**Figure S1:** Principal component analysis (PCA) of the same individuals, showing the first six principal components (PC1–PC6).
**Figure S2:** Cross‐validation (CV) error for *K* = 1–11 in the ADMIXTURE‐based population structure analysis.
**Figure S3:** Distributions of individual‐level genetic diversity metrics across the 10 chicken breeds.
**Figure S4:** Significantly enriched Gene Ontology (GO) terms for candidate genes supported by overlapping *F*_ST_ and GWAS signals.
**Figure S5:** Four comb phenotypes in chickens.

## Data Availability

The data that support the findings of this manuscript have been deposited in the GVM database (project: PRJCA043140). Information can be made available from the authors upon request; publicly available whole‐genome data for Chantecler Chicken (CTC), Emei Black Chicken (EMB), and Tibetan Chicken (TBT) were obtained from NCBI under BioProject accessions PRJNA720223, PRJNA306389, and PRJNA306389.
