# Supplementary material for: Genomic Data Reveals Key Candidate Genes Underlying Chicken Comb Morphology
Source: Evol Appl. 2026 Oct 24;19(10):e70345. doi: 10.1111/eva.70345 (PMC13695035; doi:10.1111/eva.70345)
Supplement: Supplementary file 1 [file EVA-19-e70345-s001.docx]

**Supplementary Information**

Includes Supplementary Tables S1–S8 and Figures S1–S5.

**Supplementary Table 1**

Variant counts and densities by chromosome in 228 chickens aligned to the GCA_016699485.1 reference.

| Chromosome | Length | Variants | Variants rate |
| --- | --- | --- | --- |
| 1 | 196 449 156 | 2 006 196 | 97 |
| 2 | 149 539 284 | 1 507 891 | 99 |
| 3 | 110 642 502 | 1 148 628 | 96 |
| 4 | 90 861 225 | 926 636 | 98 |
| 5 | 59 506 338 | 649 185 | 91 |
| 6 | 36 220 557 | 465 334 | 77 |
| 7 | 36 382 834 | 414 849 | 87 |
| 8 | 29 578 256 | 311 194 | 95 |
| 9 | 23 733 309 | 302 863 | 78 |
| 10 | 20 453 248 | 243 652 | 83 |
| 11 | 19 638 187 | 193 757 | 101 |
| 12 | 20 119 077 | 256 626 | 78 |
| 13 | 17 905 061 | 226 917 | 78 |
| 14 | 15 331 188 | 200 101 | 76 |
| 15 | 12 703 657 | 149 267 | 85 |
| 16 | 2 706 039 | 5 100 | 530 |
| 17 | 11 092 391 | 139 653 | 79 |
| 18 | 11 623 896 | 149 635 | 77 |
| 19 | 10 455 293 | 132 200 | 79 |
| 20 | 14 265 659 | 179 625 | 79 |
| 21 | 6 970 754 | 90 031 | 77 |
| 22 | 4 686 657 | 31 052 | 150 |
| 23 | 6 253 421 | 77 886 | 80 |
| 24 | 6 478 339 | 92 285 | 70 |
| 25 | 3 067 737 | 24 339 | 126 |
| 26 | 5 349 051 | 73 128 | 73 |
| 27 | 5 228 753 | 57 999 | 90 |
| 28 | 5 437 364 | 57 573 | 94 |
| 29 | 726 478 | 429 | 1 693 |
| 30 | 755 666 | 4 283 | 176 |
| 31 | 2 457 334 | 8 818 | 278 |
| 32 | 125 424 | 297 | 422 |
| 33 | 3 839 931 | 9 864 | 389 |
| 34 | 3 469 343 | 17 892 | 193 |
| 35 | 554 126 | 1 142 | 485 |
| 36 | 358 375 | 277 | 1 293 |
| 37 | 157 853 | 4 | 39 463 |
| 38 | 667 312 | 1 565 | 426 |
| 39 | 177 356 | 493 | 359 |
| 40 | 9 109 940 | 991 | 9 192 |
| 41 | 86 044 486 | 297 057 | 289 |
| 42 | 16 784 | 78 | 215 |
| Total | 1 041 139 641 | 10 456 792 | 99 |

**Supplementary Table 2**

Genomic distribution of sequence‐variant annotations across functional categories in chickens.

| Type (alphabetical order) | Count | Percent |
| --- | --- | --- |
| DOWNSTREAM | 9 979 818 | 8.807% |
| EXON | 1 483 286 | 1.309% |
| GENE | 1 804 | 0.002% |
| INTERGENIC | 3 273 567 | 2.889% |
| INTRON | 43 008 926 | 37.954% |
| SPLICE_SITE_ACCEPTOR | 1 672 | 0.001% |
| SPLICE_SITE_DONOR | 1 879 | 0.002% |
| SPLICE_SITE_REGION | 115 286 | 0.102% |
| TRANSCRIPT | 44 046 501 | 38.87% |
| UPSTREAM | 9 841 181 | 8.685% |
| UTR_3_PRIME | 994 096 | 0.877% |
| UTR_5_PRIME | 570 073 | 0.503% |

**Supplementary Table 3**

Functional classification and frequency distribution of variant types in the chicken cohort (n = 228).

| Type (alphabetical order) | Count | Percent |
| --- | --- | --- |
| 3_prime_UTR_variant | 994 111 | 0.876% |
| 5_prime_UTR_premature_start_codon_gain_variant | 91 384 | 0.081% |
| 5_prime_UTR_variant | 478 740 | 0.422% |
| bidirectional_gene_fusion | 924 | 0.001% |
| conservative_inframe_deletion | 731 | 0.001% |
| conservative_inframe_insertion | 299 | 0% |
| disruptive_inframe_deletion | 1 069 | 0.001% |
| disruptive_inframe_insertion | 368 | 0% |
| downstream_gene_variant | 9 979 818 | 8.798% |
| frameshift_variant | 2 509 | 0.002% |
| gene_fusion | 880 | 0.001% |
| initiator_codon_variant | 180 | 0% |
| intergenic_region | 3 273 567 | 2.886% |
| intragenic_variant | 1 085 621 | 0.957% |
| intron_variant | 43 107 286 | 38.001% |
| missense_variant | 154 179 | 0.136% |
| non_canonical_start_codon | 156 | 0% |
| non_coding_transcript_exon_variant | 791 210 | 0.697% |
| non_coding_transcript_variant | 42 960 880 | 37.872% |
| splice_acceptor_variant | 1 687 | 0.001% |
| splice_donor_variant | 1 975 | 0.002% |
| splice_region_variant | 118 771 | 0.105% |
| start_lost | 333 | 0% |
| stop_gained | 903 | 0.001% |
| stop_lost | 98 | 0% |
| stop_retained_variant | 176 | 0% |
| synonymous_variant | 547 671 | 0.483% |
| upstream_gene_variant | 9 841 181 | 8.675% |

**Supplementary Table 4**

Comparison of population genetic parameters and ANOVA results among ten breeds.

| **Breed / ANOVA Parameter** | **Inbreeding Coefficient (*F*)** | **Genetic Distance (DST)** | **Total ROH Length (Kb)** |
| --- | --- | --- | --- |
| BRC | 0.3314 (a) | 0.2113 (f) | 115517.37 (a) |
| CTC | 0.2053 (bc) | 0.1737 (g) | 120647.58 (a) |
| DHC | 0.1887 (bc) | 0.2486 (ab) | 22726.82 (cd) |
| EMB | 0.1225 (bc) | 0.2217 (e) | 7416.94 (d) |
| HTC | 0.1195 (c) | 0.2293 (de) | 11274.10 (d) |
| JHC | 0.2147 (b) | 0.2311 (d) | 66089.99 (b) |
| MQC | 0.1560 (bc) | 0.2436 (bc) | 28426.48 (cd) |
| TBT | 0.1214 (c) | 0.2504 (a) | 16266.95 (d) |
| XDC | 0.1856 (bc) | 0.2384 (c) | 47156.57 (bc) |
| ZZC | 0.1839 (bc) | 0.2394 (c) | 33900.12 (cd) |
| --- | **---** | **---** | **---** |
| ANOVA: Breed df | 9 | 9 | 9 |
| ANOVA: Residual df | 218 | 218 | 218 |
| ANOVA: Breed Sum Sq | 0.6558 | 0.05592 | 2.274×10^11^ |
| ANOVA: Residual Sum Sq | 1.2729 | 0.00935 | 2.204×10^11^ |
| ANOVA: Breed Mean Sq | 0.07287 | 0.00621 | 2.527×10^10^ |
| ANOVA: Residual Mean Sq | 0.00584 | 0.000043 | 1.011×10^9^ |
| ANOVA: F-value | 12.48 | 144.8 | 24.99 |
| ANOVA: P-value | < 0.001 | < 0.001 | < 0.001 |

Note: Data are presented as means. Different lowercase letters (a–g) within the same column indicate statistically significant differences between breeds (P < 0.05). F, inbreeding coefficient; DST, genetic distance; ROH, runs of homozygosity; df, degrees of freedom; Sum Sq, sum of squares; Mean Sq, mean squares.

**Supplementary Table 5**

Genes overlapping genomic windows in the top 1% of the F_ST_ distribution for carnation-comb versus non-carnation-comb chickens.

| Chr | Win_Start1 | Win_End1 | F_ST_ | NSNP | Gene |
| --- | --- | --- | --- | --- | --- |
| 1 | 7 030 001 | 7 040 000 | 0.08 | 222 | *CAMK1D* |
| 1 | 17 590 001 | 17 600 000 | 0.08 | 44 | *FAM19A5* |
| 1 | 21 200 001 | 21 210 000 | 0.09 | 112 | *GRM8* |
| 1 | 21 340 001 | 21 350 000 | 0.07 | 73 | *GRM8* |
| 1 | 21 350 001 | 21 360 000 | 0.07 | 78 | *GRM8* |
| 1 | 21 370 001 | 21 380 000 | 0.07 | 72 | *GRM8* |
| 1 | 21 390 001 | 21 400 000 | 0.07 | 39 | *GRM8* |
| 1 | 21 410 001 | 21 420 000 | 0.06 | 76 | *GRM8* |
| 1 | 21 420 001 | 21 430 000 | 0.12 | 54 | *GRM8* |
| 1 | 22 840 001 | 22 850 000 | 0.06 | 161 | *CADPS2* |
| 1 | 23 100 001 | 23 110 000 | 0.08 | 122 | *PTPRZ1* |
| 1 | 24 760 001 | 24 770 000 | 0.10 | 184 | *CFTR* |
| 1 | 31 010 001 | 31 020 000 | 0.07 | 159 | *SCAF11* |
| 1 | 39 440 001 | 39 450 000 | 0.06 | 117 | *SYT1* |
| 1 | 41 070 001 | 41 080 000 | 0.06 | 29 | *TMTC2* |
| 1 | 41 080 001 | 41 090 000 | 0.07 | 72 | *TMTC2* |
| 1 | 42 240 001 | 42 250 000 | 0.08 | 31 | *NTS* |
| 1 | 42 260 001 | 42 270 000 | 0.08 | 16 | *LOC427873* |
| 1 | 42 910 001 | 42 920 000 | 0.08 | 93 | *TMTC3* |
| 1 | 42 930 001 | 42 940 000 | 0.07 | 22 | *TMTC3* |
| 1 | 43 030 001 | 43 040 000 | 0.07 | 27 | *KITLG* |
| 1 | 43 040 001 | 43 050 000 | 0.07 | 28 | *KITLG* |
| 1 | 43 070 001 | 43 080 000 | 0.08 | 10 | *KITLG* |
| 1 | 44 960 001 | 44 970 000 | 0.07 | 164 | *CRADD* |
| 1 | 52 040 001 | 52 050 000 | 0.07 | 216 | *MB* |
| 1 | 57 440 001 | 57 450 000 | 0.08 | 296 | *CREB3L2* |
| 1 | 57 710 001 | 57 720 000 | 0.07 | 132 | *PTN* |
| 1 | 57 720 001 | 57 730 000 | 0.12 | 104 | *PTN* |
| 1 | 57 730 001 | 57 740 000 | 0.07 | 161 | *PTN* |
| 1 | 61 490 001 | 61 500 000 | 0.06 | 121 | *CACNA1C* |
| 1 | 62 530 001 | 62 540 000 | 0.10 | 44 | *LOC121107601* |
| 1 | 71 320 001 | 71 330 000 | 0.07 | 138 | *LOC121107607* |
| 1 | 71 320 001 | 71 330 000 | 0.07 | 138 | *PPARA* |
| 1 | 73 790 001 | 73 800 000 | 0.06 | 62 | *NDUFA9* |
| 1 | 75 520 001 | 75 530 000 | 0.06 | 23 | *TEAD4* |
| 1 | 77 530 001 | 77 540 000 | 0.07 | 170 | *FAM131B* |
| 1 | 83 050 001 | 83 060 000 | 0.08 | 196 | *CCDC191* |
| 1 | 83 880 001 | 83 890 000 | 0.07 | 101 | *F5* |
| 1 | 85 820 001 | 85 830 000 | 0.10 | 79 | *ZPLD1* |
| 1 | 85 830 001 | 85 840 000 | 0.06 | 107 | *ZPLD1* |
| 1 | 92 610 001 | 92 620 000 | 0.08 | 198 | *GJA5* |
| 1 | 95 360 001 | 95 370 000 | 0.07 | 125 | *GBE1* |
| 1 | 96 400 001 | 96 410 000 | 0.07 | 74 | *ROBO1* |
| 1 | 97 320 001 | 97 330 000 | 0.07 | 25 | *ROBO2* |
| 1 | 97 330 001 | 97 340 000 | 0.11 | 43 | *ROBO2* |
| 1 | 101 000 000 | 101 000 000 | 0.07 | 18 | *NCAM2* |
| 1 | 103 000 000 | 103 000 000 | 0.10 | 143 | *CYYR1* |
| 1 | 115 000 000 | 115 000 000 | 0.07 | 78 | *DMD* |
| 1 | 115 000 000 | 115 000 000 | 0.08 | 19 | *DMD* |
| 1 | 119 000 000 | 119 000 000 | 0.09 | 50 | *CNKSR2* |
| 1 | 120 000 000 | 120 000 000 | 0.07 | 115 | *SH3KBP1* |
| 1 | 121 000 000 | 121 000 000 | 0.07 | 127 | *CDKL5* |
| 1 | 121 000 000 | 121 000 000 | 0.06 | 199 | *CDKL5* |
| 1 | 127 000 000 | 127 000 000 | 0.06 | 36 | *STS* |
| 1 | 127 000 000 | 127 000 000 | 0.06 | 32 | *NLGN4Y* |
| 1 | 130 000 000 | 130 000 000 | 0.08 | 153 | *PPP2R3B* |
| 1 | 130 000 000 | 130 000 000 | 0.06 | 250 | *STAG3* |
| 1 | 130 000 000 | 130 000 000 | 0.06 | 163 | *TUBGCP5* |
| 1 | 131 000 000 | 131 000 000 | 0.07 | 142 | *HERC2* |
| 1 | 131 000 000 | 131 000 000 | 0.07 | 64 | *GABRG3* |
| 1 | 131 000 000 | 131 000 000 | 0.08 | 112 | *GABRG3* |
| 1 | 131 000 000 | 131 000 000 | 0.07 | 94 | *GABRG3* |
| 1 | 131 000 000 | 131 000 000 | 0.07 | 86 | *GABRG3* |
| 1 | 131 000 000 | 131 000 000 | 0.07 | 124 | *GABRG3* |
| 1 | 131 000 000 | 131 000 000 | 0.10 | 145 | *GABRG3* |
| 1 | 131 000 000 | 131 000 000 | 0.13 | 121 | *GABRG3* |
| 1 | 131 000 000 | 131 000 000 | 0.09 | 141 | *GABRG3* |
| 1 | 131 000 000 | 131 000 000 | 0.08 | 156 | *GABRG3* |
| 1 | 131 000 000 | 131 000 000 | 0.06 | 171 | *GABRG3* |
| 1 | 131 000 000 | 131 000 000 | 0.07 | 149 | *GABRG3* |
| 1 | 131 000 000 | 131 000 000 | 0.07 | 144 | *GABRG3* |
| 1 | 131 000 000 | 131 000 000 | 0.07 | 179 | *GABRB3* |
| 1 | 133 000 000 | 133 000 000 | 0.07 | 230 | *CRACDL* |
| 1 | 133 000 000 | 133 000 000 | 0.07 | 111 | *REV1* |
| 1 | 134 000 000 | 134 000 000 | 0.07 | 139 | *IL1RL1* |
| 1 | 134 000 000 | 134 000 000 | 0.08 | 118 | *IL1RL1* |
| 1 | 135 000 000 | 135 000 000 | 0.07 | 183 | *FHL2* |
| 1 | 159 000 000 | 159 000 000 | 0.06 | 12 | *PCDH9* |
| 1 | 159 000 000 | 159 000 000 | 0.08 | 37 | *PCDH9* |
| 1 | 160 000 000 | 160 000 000 | 0.07 | 26 | *PCDH9* |
| 1 | 160 000 000 | 160 000 000 | 0.08 | 25 | *PCDH9* |
| 1 | 162 000 000 | 162 000 000 | 0.07 | 94 | *DIAPH3* |
| 1 | 169 000 000 | 169 000 000 | 0.06 | 198 | *KPNA3* |
| 1 | 170 000 000 | 170 000 000 | 0.09 | 149 | *INTS6* |
| 1 | 171 000 000 | 171 000 000 | 0.08 | 248 | *LHFPL1* |
| 1 | 172 000 000 | 172 000 000 | 0.09 | 86 | *TRPC4* |
| 1 | 174 000 000 | 174 000 000 | 0.07 | 284 | *STARD13* |
| 1 | 174 000 000 | 174 000 000 | 0.07 | 281 | *STARD13* |
| 1 | 174 000 000 | 174 000 000 | 0.09 | 213 | *STARD13* |
| 1 | 174 000 000 | 174 000 000 | 0.08 | 160 | *KL* |
| 1 | 176 000 000 | 176 000 000 | 0.07 | 157 | *MTUS2* |
| 1 | 176 000 000 | 176 000 000 | 0.10 | 278 | *MTUS2* |
| 1 | 176 000 000 | 176 000 000 | 0.09 | 184 | *MTUS2* |
| 1 | 176 000 000 | 176 000 000 | 0.08 | 253 | *MTUS2* |
| 1 | 176 000 000 | 176 000 000 | 0.08 | 237 | *WASF3* |
| 1 | 177 000 000 | 177 000 000 | 0.06 | 148 | *SACS* |
| 1 | 178 000 000 | 178 000 000 | 0.07 | 90 | *SACS* |
| 1 | 178 000 000 | 178 000 000 | 0.10 | 205 | *SGCG* |
| 1 | 178 000 000 | 178 000 000 | 0.09 | 205 | *SGCG* |
| 1 | 178 000 000 | 178 000 000 | 0.07 | 162 | *SGCG* |
| 1 | 178 000 000 | 178 000 000 | 0.09 | 127 | *SGCG* |
| 1 | 178 000 000 | 178 000 000 | 0.07 | 257 | *ZDHHC20* |
| 1 | 179 000 000 | 179 000 000 | 0.07 | 234 | *MPHOSPH8* |
| 1 | 179 000 000 | 179 000 000 | 0.07 | 130 | *MPHOSPH8* |
| 1 | 179 000 000 | 179 000 000 | 0.07 | 149 | *MPHOSPH8* |
| 1 | 179 000 000 | 179 000 000 | 0.07 | 149 | *PARP4* |
| 1 | 179 000 000 | 179 000 000 | 0.10 | 51 | *PARP4* |
| 1 | 179 000 000 | 179 000 000 | 0.07 | 85 | *PARP4* |
| 1 | 179 000 000 | 179 000 000 | 0.09 | 104 | *PARP4* |
| 1 | 179 000 000 | 179 000 000 | 0.07 | 293 | *RNF17* |
| 1 | 179 000 000 | 179 000 000 | 0.09 | 233 | *RNF17* |
| 1 | 179 000 000 | 179 000 000 | 0.07 | 316 | *RNF17* |
| 1 | 182 000 000 | 182 000 000 | 0.07 | 174 | *PDGFD* |
| 1 | 183 000 000 | 183 000 000 | 0.07 | 192 | *ARHGAP42* |
| 1 | 183 000 000 | 183 000 000 | 0.06 | 143 | *ARHGAP42* |
| 1 | 191 000 000 | 191 000 000 | 0.16 | 34 | *LOC101747503* |
| 1 | 192 000 000 | 192 000 000 | 0.07 | 75 | *TENM4* |
| 1 | 192 000 000 | 192 000 000 | 0.08 | 157 | *TENM4* |
| 2 | 16 290 001 | 16 300 000 | 0.07 | 225 | *MYO3A* |
| 2 | 17 070 001 | 17 080 000 | 0.07 | 144 | *KIAA1217* |
| 2 | 19 120 001 | 19 130 000 | 0.07 | 191 | *CACNB2* |
| 2 | 22 400 001 | 22 410 000 | 0.07 | 216 | *CYP51A1* |
| 2 | 22 470 001 | 22 480 000 | 0.07 | 116 | *ANKIB1* |
| 2 | 22 530 001 | 22 540 000 | 0.07 | 220 | *LOC420552* |
| 2 | 22 530 001 | 22 540 000 | 0.07 | 220 | *ACCSL* |
| 2 | 25 910 001 | 25 920 000 | 0.07 | 86 | *NDUFA4* |
| 2 | 26 200 001 | 26 210 000 | 0.07 | 70 | *THSD7A* |
| 2 | 26 210 001 | 26 220 000 | 0.11 | 46 | *THSD7A* |
| 2 | 26 400 001 | 26 410 000 | 0.06 | 56 | *THSD7A* |
| 2 | 27 210 001 | 27 220 000 | 0.07 | 74 | *ETV1* |
| 2 | 27 510 001 | 27 520 000 | 0.08 | 50 | *DGKB* |
| 2 | 27 570 001 | 27 580 000 | 0.08 | 27 | *DGKB* |
| 2 | 27 580 001 | 27 590 000 | 0.15 | 51 | *DGKB* |
| 2 | 27 590 001 | 27 600 000 | 0.13 | 31 | *DGKB* |
| 2 | 27 600 001 | 27 610 000 | 0.08 | 78 | *DGKB* |
| 2 | 27 610 001 | 27 620 000 | 0.09 | 43 | *DGKB* |
| 2 | 27 910 001 | 27 920 000 | 0.06 | 93 | *AGMO* |
| 2 | 28 210 001 | 28 220 000 | 0.06 | 124 | *ISPD* |
| 2 | 28 220 001 | 28 230 000 | 0.07 | 119 | *ISPD* |
| 2 | 28 260 001 | 28 270 000 | 0.08 | 74 | *ISPD* |
| 2 | 28 290 001 | 28 300 000 | 0.07 | 146 | *ISPD* |
| 2 | 28 370 001 | 28 380 000 | 0.07 | 189 | *ANKMY2* |
| 2 | 29 650 001 | 29 660 000 | 0.08 | 117 | *TWISTNB* |
| 2 | 29 800 001 | 29 810 000 | 0.09 | 143 | *MACC1* |
| 2 | 29 810 001 | 29 820 000 | 0.13 | 73 | *MACC1* |
| 2 | 29 820 001 | 29 830 000 | 0.07 | 114 | *MACC1* |
| 2 | 29 900 001 | 29 910 000 | 0.07 | 83 | *ITGB8* |
| 2 | 39 150 001 | 39 160 000 | 0.09 | 142 | *RBMS3* |
| 2 | 41 450 001 | 41 460 000 | 0.10 | 201 | *COL6A6* |
| 2 | 49 640 001 | 49 650 000 | 0.06 | 86 | *POU6F2* |
| 2 | 49 710 001 | 49 720 000 | 0.07 | 75 | *POU6F2* |
| 2 | 49 730 001 | 49 740 000 | 0.09 | 79 | *POU6F2* |
| 2 | 53 950 001 | 53 960 000 | 0.07 | 106 | *CUL1* |
| 2 | 53 950 001 | 53 960 000 | 0.07 | 106 | *EZH2* |
| 2 | 53 990 001 | 54 000 000 | 0.07 | 99 | *EZH2* |
| 2 | 54 140 001 | 54 150 000 | 0.07 | 230 | *RAMP3* |
| 2 | 54 150 001 | 54 160 000 | 0.09 | 110 | *RAMP3* |
| 2 | 54 170 001 | 54 180 000 | 0.10 | 135 | *RAMP3* |
| 2 | 54 180 001 | 54 190 000 | 0.12 | 181 | *RAMP3* |
| 2 | 54 190 001 | 54 200 000 | 0.10 | 124 | *RAMP3* |
| 2 | 54 460 001 | 54 470 000 | 0.06 | 137 | *ADCY1* |
| 2 | 55 180 001 | 55 190 000 | 0.06 | 157 | *SLC12A7* |
| 2 | 55 400 001 | 55 410 000 | 0.07 | 78 | *SLC9A3* |
| 2 | 55 410 001 | 55 420 000 | 0.07 | 41 | *SLC9A3* |
| 2 | 55 440 001 | 55 450 000 | 0.10 | 71 | *SLC9A3* |
| 2 | 73 920 001 | 73 930 000 | 0.07 | 119 | *CDH18* |
| 2 | 73 930 001 | 73 940 000 | 0.07 | 105 | *CDH18* |
| 2 | 77 440 001 | 77 450 000 | 0.07 | 216 | *CTNND2* |
| 2 | 80 800 001 | 80 810 000 | 0.07 | 235 | *COBL* |
| 2 | 80 820 001 | 80 830 000 | 0.07 | 231 | *COBL* |
| 2 | 93 370 001 | 93 380 000 | 0.08 | 40 | *DOK6* |
| 2 | 93 400 001 | 93 410 000 | 0.13 | 47 | *DOK6* |
| 2 | 93 410 001 | 93 420 000 | 0.11 | 30 | *DOK6* |
| 2 | 93 420 001 | 93 430 000 | 0.07 | 35 | *DOK6* |
| 2 | 93 450 001 | 93 460 000 | 0.08 | 19 | *DOK6* |
| 2 | 93 460 001 | 93 470 000 | 0.08 | 41 | *DOK6* |
| 2 | 93 470 001 | 93 480 000 | 0.07 | 74 | *DOK6* |
| 2 | 93 480 001 | 93 490 000 | 0.07 | 55 | *DOK6* |
| 2 | 93 490 001 | 93 500 000 | 0.08 | 70 | *DOK6* |
| 2 | 113 000 000 | 113 000 000 | 0.06 | 122 | *NKAIN3* |
| 2 | 113 000 000 | 113 000 000 | 0.06 | 129 | *NKAIN3* |
| 2 | 116 000 000 | 116 000 000 | 0.06 | 137 | *NCOA2* |
| 2 | 127 000 000 | 127 000 000 | 0.06 | 139 | *STK3* |
| 2 | 146 000 000 | 146 000 000 | 0.06 | 100 | *TSNARE1* |
| 2 | 146 000 000 | 146 000 000 | 0.06 | 143 | *TSNARE1* |
| 2 | 149 000 000 | 149 000 000 | 0.09 | 12 | *LOC121109775* |
| 3 | 5 420 001 | 5 430 000 | 0.07 | 211 | *MACROD2* |
| 3 | 7 290 001 | 7 300 000 | 0.07 | 149 | *NRXN1* |
| 3 | 25 360 001 | 25 370 000 | 0.08 | 37 | *CAMKMT* |
| 3 | 26 010 001 | 26 020 000 | 0.07 | 109 | *PRKCE* |
| 3 | 28 460 001 | 28 470 000 | 0.07 | 120 | *DAAM2* |
| 3 | 28 490 001 | 28 500 000 | 0.06 | 84 | *DAAM2* |
| 3 | 28 500 001 | 28 510 000 | 0.07 | 112 | *DAAM2* |
| 3 | 28 700 001 | 28 710 000 | 0.07 | 37 | *KIF6* |
| 3 | 28 720 001 | 28 730 000 | 0.07 | 63 | *KIF6* |
| 3 | 30 490 001 | 30 500 000 | 0.06 | 165 | *RSPH9* |
| 3 | 31 700 001 | 31 710 000 | 0.07 | 268 | *HEATR5B* |
| 3 | 34 140 001 | 34 150 000 | 0.06 | 169 | *KIF26B* |
| 3 | 35 650 001 | 35 660 000 | 0.07 | 92 | *RGS7* |
| 3 | 50 020 001 | 50 030 000 | 0.07 | 131 | *SCAF8* |
| 3 | 51 410 001 | 51 420 000 | 0.07 | 156 | *SNX9* |
| 3 | 53 800 001 | 53 810 000 | 0.09 | 172 | *REPS1* |
| 3 | 53 810 001 | 53 820 000 | 0.10 | 185 | *REPS1* |
| 3 | 53 820 001 | 53 830 000 | 0.08 | 77 | *REPS1* |
| 3 | 53 830 001 | 53 840 000 | 0.06 | 46 | *REPS1* |
| 3 | 53 840 001 | 53 850 000 | 0.11 | 134 | *REPS1* |
| 3 | 55 320 001 | 55 330 000 | 0.07 | 104 | *AHI1* |
| 3 | 57 830 001 | 57 840 000 | 0.08 | 44 | *LAMA2* |
| 3 | 58 580 001 | 58 590 000 | 0.08 | 75 | *PTPRK* |
| 3 | 58 590 001 | 58 600 000 | 0.07 | 57 | *PTPRK* |
| 3 | 58 600 001 | 58 610 000 | 0.07 | 102 | *PTPRK* |
| 3 | 62 370 001 | 62 380 000 | 0.09 | 49 | *FAM184A* |
| 3 | 63 250 001 | 63 260 000 | 0.06 | 158 | *VGLL2* |
| 3 | 63 510 001 | 63 520 000 | 0.07 | 220 | *RWDD1* |
| 3 | 63 510 001 | 63 520 000 | 0.07 | 220 | *FAM26D* |
| 3 | 63 520 001 | 63 530 000 | 0.07 | 137 | *TRAPPC3L* |
| 3 | 63 740 001 | 63 750 000 | 0.06 | 95 | *FRK* |
| 3 | 66 290 001 | 66 300 000 | 0.07 | 192 | *METTL24* |
| 3 | 67 140 001 | 67 150 000 | 0.09 | 215 | *AFG1L* |
| 3 | 67 430 001 | 67 440 000 | 0.08 | 251 | *SOBP* |
| 3 | 74 790 001 | 74 800 000 | 0.09 | 53 | *MAP3K7* |
| 3 | 75 600 001 | 75 610 000 | 0.07 | 202 | *RNGTT* |
| 3 | 75 780 001 | 75 790 000 | 0.07 | 153 | *LOC101749895* |
| 3 | 76 230 001 | 76 240 000 | 0.08 | 116 | *ZNF292* |
| 3 | 84 450 001 | 84 460 000 | 0.08 | 13 | *LOC121110182* |
| 3 | 84 470 001 | 84 480 000 | 0.09 | 17 | *LOC121110182* |
| 3 | 84 530 001 | 84 540 000 | 0.07 | 52 | *LOC121110182* |
| 3 | 84 640 001 | 84 650 000 | 0.07 | 41 | *EYS* |
| 3 | 90 910 001 | 90 920 000 | 0.07 | 83 | *DLGAP2* |
| 3 | 99 300 001 | 99 310 000 | 0.07 | 41 | *FAM49A* |
| 3 | 111 000 000 | 111 000 000 | 0.06 | 45 | *EVA1A* |
| 4 | 250 001 | 260 000 | 0.07 | 90 | *HSF3* |
| 4 | 250 001 | 260 000 | 0.07 | 90 | *HEPH* |
| 4 | 260 001 | 270 000 | 0.11 | 145 | *HEPH* |
| 4 | 270 001 | 280 000 | 0.12 | 151 | *HEPH* |
| 4 | 5 880 001 | 5 890 000 | 0.08 | 48 | *DIAPH2* |
| 4 | 15 450 001 | 15 460 000 | 0.06 | 140 | *TENM1* |
| 4 | 17 230 001 | 17 240 000 | 0.07 | 88 | *NRK* |
| 4 | 19 500 001 | 19 510 000 | 0.07 | 24 | *TLR2* |
| 4 | 23 950 001 | 23 960 000 | 0.07 | 136 | *SPOCK3* |
| 4 | 32 640 001 | 32 650 000 | 0.07 | 70 | *LRBA* |
| 4 | 32 650 001 | 32 660 000 | 0.07 | 69 | *LRBA* |
| 4 | 34 660 001 | 34 670 000 | 0.06 | 251 | *HERC3* |
| 4 | 34 660 001 | 34 670 000 | 0.06 | 251 | *FAM13A* |
| 4 | 35 200 001 | 35 210 000 | 0.07 | 153 | *CCSER1* |
| 4 | 35 250 001 | 35 260 000 | 0.06 | 120 | *CCSER1* |
| 4 | 35 370 001 | 35 380 000 | 0.08 | 94 | *CCSER1* |
| 4 | 35 750 001 | 35 760 000 | 0.07 | 24 | *CCSER1* |
| 4 | 36 140 001 | 36 150 000 | 0.09 | 41 | *GRID2* |
| 4 | 36 150 001 | 36 160 000 | 0.06 | 57 | *GRID2* |
| 4 | 36 170 001 | 36 180 000 | 0.07 | 46 | *GRID2* |
| 4 | 36 180 001 | 36 190 000 | 0.10 | 74 | *GRID2* |
| 4 | 36 230 001 | 36 240 000 | 0.07 | 100 | *GRID2* |
| 4 | 55 680 001 | 55 690 000 | 0.06 | 79 | *NDST4* |
| 4 | 56 710 001 | 56 720 000 | 0.07 | 222 | *AP1AR* |
| 4 | 60 390 001 | 60 400 000 | 0.07 | 184 | *MANBA* |
| 4 | 61 270 001 | 61 280 000 | 0.07 | 57 | *FAT1* |
| 4 | 63 170 001 | 63 180 000 | 0.09 | 53 | *TUSC3* |
| 4 | 63 220 001 | 63 230 000 | 0.09 | 41 | *TUSC3* |
| 4 | 63 280 001 | 63 290 000 | 0.07 | 51 | *TUSC3* |
| 4 | 63 390 001 | 63 400 000 | 0.07 | 81 | *SGCZ* |
| 4 | 63 410 001 | 63 420 000 | 0.07 | 66 | *SGCZ* |
| 4 | 63 420 001 | 63 430 000 | 0.07 | 56 | *SGCZ* |
| 4 | 63 510 001 | 63 520 000 | 0.08 | 47 | *SGCZ* |
| 4 | 63 630 001 | 63 640 000 | 0.08 | 31 | *SGCZ* |
| 4 | 63 650 001 | 63 660 000 | 0.07 | 34 | *SGCZ* |
| 4 | 63 730 001 | 63 740 000 | 0.07 | 42 | *SGCZ* |
| 4 | 64 000 001 | 64 010 000 | 0.08 | 94 | *DLC1* |
| 4 | 64 020 001 | 64 030 000 | 0.09 | 71 | *DLC1* |
| 4 | 68 560 001 | 68 570 000 | 0.08 | 88 | *N4BP2* |
| 4 | 79 990 001 | 80 000 000 | 0.07 | 57 | *SORCS2* |
| 5 | 2 320 001 | 2 330 000 | 0.12 | 54 | *PRMT3* |
| 5 | 2 330 001 | 2 340 000 | 0.10 | 37 | *PRMT3* |
| 5 | 2 340 001 | 2 350 000 | 0.08 | 68 | *PRMT3* |
| 5 | 2 460 001 | 2 470 000 | 0.07 | 36 | *NELL1* |
| 5 | 2 470 001 | 2 480 000 | 0.08 | 23 | *NELL1* |
| 5 | 2 510 001 | 2 520 000 | 0.07 | 16 | *NELL1* |
| 5 | 2 520 001 | 2 530 000 | 0.07 | 23 | *NELL1* |
| 5 | 7 000 001 | 7 010 000 | 0.06 | 117 | *PPFIBP2* |
| 5 | 7 010 001 | 7 020 000 | 0.07 | 69 | *PPFIBP2* |
| 5 | 7 020 001 | 7 030 000 | 0.09 | 135 | *PPFIBP2* |
| 5 | 7 060 001 | 7 070 000 | 0.08 | 152 | *PPFIBP2* |
| 5 | 7 060 001 | 7 070 000 | 0.08 | 152 | *CYB5R2* |
| 5 | 10 180 001 | 10 190 000 | 0.07 | 92 | *PDE3B* |
| 5 | 10 180 001 | 10 190 000 | 0.07 | 92 | *CALCA* |
| 5 | 10 800 001 | 10 810 000 | 0.07 | 101 | *SOX6* |
| 5 | 13 020 001 | 13 030 000 | 0.07 | 131 | *KCNQ1* |
| 5 | 14 330 001 | 14 340 000 | 0.06 | 35 | *MUC5L* |
| 5 | 20 160 001 | 20 170 000 | 0.07 | 54 | *LRRC4C* |
| 5 | 20 730 001 | 20 740 000 | 0.07 | 141 | *LOC112532543* |
| 5 | 24 780 001 | 24 790 000 | 0.07 | 97 | *SPTBN5* |
| 5 | 28 150 001 | 28 160 000 | 0.07 | 132 | *RAD51B* |
| 5 | 30 730 001 | 30 740 000 | 0.07 | 126 | *MEIS2* |
| 5 | 34 410 001 | 34 420 000 | 0.06 | 25 | *AKAP6* |
| 5 | 34 980 001 | 34 990 000 | 0.07 | 15 | *NPAS3* |
| 5 | 34 990 001 | 35 000 000 | 0.07 | 52 | *NPAS3* |
| 5 | 40 310 001 | 40 320 000 | 0.06 | 23 | *TSHR* |
| 5 | 43 130 001 | 43 140 000 | 0.06 | 269 | *EFCAB11* |
| 5 | 45 860 001 | 45 870 000 | 0.06 | 134 | *BDKRB2* |
| 5 | 48 870 001 | 48 880 000 | 0.07 | 240 | *PPP2R5C* |
| 5 | 48 910 001 | 48 920 000 | 0.06 | 165 | *PPP2R5C* |
| 5 | 48 960 001 | 48 970 000 | 0.06 | 285 | *DYNC1H1* |
| 5 | 52 540 001 | 52 550 000 | 0.06 | 165 | *ESR2* |
| 5 | 52 690 001 | 52 700 000 | 0.08 | 109 | *SYNE2* |
| 6 | 4 450 001 | 4 460 000 | 0.07 | 240 | *NRG3* |
| 6 | 5 030 001 | 5 040 000 | 0.07 | 223 | *TSPAN14* |
| 6 | 11 350 001 | 11 360 000 | 0.09 | 135 | *RUFY2* |
| 6 | 11 360 001 | 11 370 000 | 0.08 | 238 | *RUFY2* |
| 6 | 11 360 001 | 11 370 000 | 0.08 | 238 | *DNA2* |
| 6 | 11 370 001 | 11 380 000 | 0.07 | 203 | *DNA2* |
| 6 | 11 500 001 | 11 510 000 | 0.06 | 130 | *CCAR1* |
| 6 | 11 510 001 | 11 520 000 | 0.08 | 182 | *CCAR1* |
| 6 | 11 520 001 | 11 530 000 | 0.09 | 330 | *STOX1* |
| 6 | 11 530 001 | 11 540 000 | 0.08 | 267 | *STOX1* |
| 6 | 11 530 001 | 11 540 000 | 0.08 | 267 | *DDX21* |
| 6 | 12 260 001 | 12 270 000 | 0.07 | 229 | *ADAMTS14* |
| 6 | 12 320 001 | 12 330 000 | 0.07 | 146 | *SGPL1* |
| 6 | 12 320 001 | 12 330 000 | 0.07 | 146 | *PCBD1* |
| 6 | 14 520 001 | 14 530 000 | 0.07 | 13 | *KCNMA1* |
| 6 | 15 790 001 | 15 800 000 | 0.07 | 67 | *KAT6B* |
| 6 | 16 500 001 | 16 510 000 | 0.06 | 213 | *NDST2* |
| 6 | 25 220 001 | 25 230 000 | 0.08 | 180 | *SORCS3* |
| 6 | 28 300 001 | 28 310 000 | 0.06 | 192 | *CASP7* |
| 6 | 34 100 001 | 34 110 000 | 0.06 | 284 | *PTPRE* |
| 6 | 35 920 001 | 35 930 000 | 0.06 | 96 | *INPP5A* |
| 7 | 6 240 001 | 6 250 000 | 0.07 | 109 | *HDAC4* |
| 7 | 9 680 001 | 9 690 000 | 0.06 | 258 | *ANKRD44* |
| 7 | 12 040 001 | 12 050 000 | 0.06 | 135 | *DYTN* |
| 7 | 12 040 001 | 12 050 000 | 0.06 | 135 | *FAM237A* |
| 7 | 14 780 001 | 14 790 000 | 0.08 | 48 | *TTN* |
| 7 | 14 900 001 | 14 910 000 | 0.08 | 13 | *TTN* |
| 7 | 14 970 001 | 14 980 000 | 0.07 | 42 | *TTN* |
| 7 | 14 990 001 | 15 000 000 | 0.06 | 62 | *TTN* |
| 7 | 15 070 001 | 15 080 000 | 0.06 | 118 | *OSBPL6* |
| 7 | 15 150 001 | 15 160 000 | 0.07 | 204 | *RBM45* |
| 7 | 15 180 001 | 15 190 000 | 0.07 | 115 | *PDE11A* |
| 7 | 15 430 001 | 15 440 000 | 0.07 | 250 | *NFE2L2* |
| 7 | 15 440 001 | 15 450 000 | 0.08 | 162 | *NFE2L2* |
| 7 | 15 440 001 | 15 450 000 | 0.08 | 162 | *HNRNPA3* |
| 7 | 17 480 001 | 17 490 000 | 0.08 | 236 | *METAP1D* |
| 7 | 17 560 001 | 17 570 000 | 0.07 | 88 | *SLC25A12* |
| 7 | 17 620 001 | 17 630 000 | 0.07 | 40 | *CYBRD1* |
| 7 | 17 910 001 | 17 920 000 | 0.06 | 104 | *MYO3B* |
| 7 | 18 020 001 | 18 030 000 | 0.07 | 54 | *MYO3B* |
| 7 | 18 030 001 | 18 040 000 | 0.07 | 103 | *MYO3B* |
| 7 | 18 130 001 | 18 140 000 | 0.07 | 117 | *UBR3* |
| 7 | 18 140 001 | 18 150 000 | 0.09 | 134 | *UBR3* |
| 7 | 18 150 001 | 18 160 000 | 0.08 | 58 | *UBR3* |
| 7 | 18 280 001 | 18 290 000 | 0.06 | 178 | *FASTKD1* |
| 7 | 18 280 001 | 18 290 000 | 0.06 | 178 | *KLHL41* |
| 7 | 18 300 001 | 18 310 000 | 0.07 | 196 | *BBS5* |
| 7 | 18 300 001 | 18 310 000 | 0.07 | 196 | *LOC424167* |
| 7 | 18 310 001 | 18 320 000 | 0.06 | 157 | *LOC424167* |
| 7 | 18 400 001 | 18 410 000 | 0.08 | 86 | *LRP2* |
| 7 | 18 410 001 | 18 420 000 | 0.09 | 71 | *LRP2* |
| 7 | 18 440 001 | 18 450 000 | 0.09 | 76 | *LRP2* |
| 7 | 18 460 001 | 18 470 000 | 0.06 | 101 | *LRP2* |
| 7 | 18 460 001 | 18 470 000 | 0.06 | 101 | *LOC121106449* |
| 7 | 18 470 001 | 18 480 000 | 0.07 | 150 | *LOC121106449* |
| 7 | 18 480 001 | 18 490 000 | 0.08 | 128 | *LOC121106449* |
| 7 | 18 470 001 | 18 480 000 | 0.07 | 150 | *ABCB11* |
| 7 | 18 480 001 | 18 490 000 | 0.08 | 128 | *ABCB11* |
| 7 | 18 540 001 | 18 550 000 | 0.07 | 97 | *G6PC2* |
| 7 | 18 550 001 | 18 560 000 | 0.08 | 91 | *SPC25* |
| 7 | 18 570 001 | 18 580 000 | 0.07 | 81 | *CERS6* |
| 7 | 18 590 001 | 18 600 000 | 0.07 | 56 | *CERS6* |
| 7 | 18 610 001 | 18 620 000 | 0.10 | 103 | *CERS6* |
| 7 | 18 620 001 | 18 630 000 | 0.10 | 101 | *CERS6* |
| 7 | 18 630 001 | 18 640 000 | 0.06 | 102 | *CERS6* |
| 7 | 18 670 001 | 18 680 000 | 0.08 | 155 | *CERS6* |
| 7 | 18 700 001 | 18 710 000 | 0.06 | 161 | *STK39* |
| 7 | 18 710 001 | 18 720 000 | 0.06 | 172 | *STK39* |
| 7 | 18 730 001 | 18 740 000 | 0.09 | 74 | *STK39* |
| 7 | 18 770 001 | 18 780 000 | 0.06 | 48 | *STK39* |
| 7 | 18 860 001 | 18 870 000 | 0.08 | 81 | *B3GALT1* |
| 7 | 18 900 001 | 18 910 000 | 0.06 | 69 | *B3GALT1* |
| 7 | 18 910 001 | 18 920 000 | 0.06 | 83 | *B3GALT1* |
| 7 | 18 970 001 | 18 980 000 | 0.07 | 96 | *B3GALT1* |
| 7 | 19 010 001 | 19 020 000 | 0.08 | 126 | *XIRP2* |
| 7 | 19 020 001 | 19 030 000 | 0.06 | 71 | *XIRP2* |
| 7 | 19 030 001 | 19 040 000 | 0.08 | 44 | *XIRP2* |
| 7 | 19 100 001 | 19 110 000 | 0.09 | 115 | *SCN9A* |
| 7 | 19 200 001 | 19 210 000 | 0.06 | 41 | *SCN1A* |
| 7 | 19 580 001 | 19 590 000 | 0.07 | 26 | *SCN3A* |
| 7 | 19 640 001 | 19 650 000 | 0.08 | 79 | *SLC38A11* |
| 7 | 20 480 001 | 20 490 000 | 0.07 | 57 | *KCNH7* |
| 7 | 20 500 001 | 20 510 000 | 0.06 | 79 | *KCNH7* |
| 7 | 20 520 001 | 20 530 000 | 0.06 | 43 | *KCNH7* |
| 7 | 20 720 001 | 20 730 000 | 0.07 | 19 | *FAP* |
| 7 | 20 850 001 | 20 860 000 | 0.07 | 43 | *SLC4A10* |
| 7 | 21 040 001 | 21 050 000 | 0.08 | 110 | *PSMD14* |
| 7 | 21 040 001 | 21 050 000 | 0.08 | 110 | *RBMS1* |
| 7 | 21 080 001 | 21 090 000 | 0.06 | 65 | *RBMS1* |
| 7 | 21 090 001 | 21 100 000 | 0.07 | 45 | *RBMS1* |
| 7 | 21 110 001 | 21 120 000 | 0.06 | 79 | *RBMS1* |
| 7 | 21 080 001 | 21 090 000 | 0.06 | 65 | *TANK* |
| 7 | 21 090 001 | 21 100 000 | 0.07 | 45 | *TANK* |
| 7 | 21 480 001 | 21 490 000 | 0.08 | 273 | *PLA2R1* |
| 7 | 21 610 001 | 21 620 000 | 0.06 | 269 | *MYO10L* |
| 8 | 910 001 | 920 000 | 0.07 | 87 | *NTNG1* |
| 8 | 2 630 001 | 2 640 000 | 0.07 | 148 | *ASPM* |
| 8 | 23 040 001 | 23 050 000 | 0.08 | 61 | *BEND5* |
| 8 | 28 110 001 | 28 120 000 | 0.08 | 240 | *PDE4B* |
| 9 | 500 001 | 510 000 | 0.07 | 26 | *AIRE* |
| 9 | 8 220 001 | 8 230 000 | 0.07 | 157 | *DOCK10* |
| 9 | 8 230 001 | 8 240 000 | 0.06 | 145 | *DOCK10* |
| 9 | 9 080 001 | 9 090 000 | 0.06 | 150 | *COL4A3* |
| 9 | 12 910 001 | 12 920 000 | 0.06 | 72 | *FCGBP* |
| 10 | 1 950 001 | 1 960 000 | 0.07 | 221 | *NEO1* |
| 10 | 1 960 001 | 1 970 000 | 0.07 | 247 | *NEO1* |
| 10 | 3 660 001 | 3 670 000 | 0.08 | 303 | *CHRNA5* |
| 10 | 3 660 001 | 3 670 000 | 0.08 | 303 | *PSMA4* |
| 10 | 4 000 001 | 4 010 000 | 0.06 | 88 | *HERC1* |
| 10 | 4 040 001 | 4 050 000 | 0.07 | 123 | *HERC1* |
| 10 | 4 050 001 | 4 060 000 | 0.07 | 199 | *HERC1* |
| 10 | 4 050 001 | 4 060 000 | 0.07 | 199 | *FBXL22* |
| 10 | 4 050 001 | 4 060 000 | 0.07 | 199 | *USP3* |
| 10 | 4 530 001 | 4 540 000 | 0.07 | 64 | *VPS13C* |
| 10 | 6 090 001 | 6 100 000 | 0.07 | 249 | *TJP1* |
| 10 | 6 100 001 | 6 110 000 | 0.06 | 187 | *TJP1* |
| 10 | 6 190 001 | 6 200 000 | 0.07 | 137 | *TJP1* |
| 10 | 6 570 001 | 6 580 000 | 0.06 | 155 | *THSD4* |
| 10 | 6 600 001 | 6 610 000 | 0.06 | 94 | *THSD4* |
| 10 | 7 570 001 | 7 580 000 | 0.07 | 211 | *TCF12* |
| 10 | 8 010 001 | 8 020 000 | 0.07 | 219 | *RAB27A* |
| 10 | 16 650 001 | 16 660 000 | 0.07 | 96 | *FAM169B* |
| 10 | 17 050 001 | 17 060 000 | 0.08 | 145 | *MEF2A* |
| 10 | 18 540 001 | 18 550 000 | 0.08 | 209 | *ZWILCH* |
| 10 | 18 540 001 | 18 550 000 | 0.08 | 209 | *LCTL* |
| 11 | 4 540 001 | 4 550 000 | 0.07 | 66 | *RPGRIP1L* |
| 11 | 4 550 001 | 4 560 000 | 0.08 | 54 | *RPGRIP1L* |
| 11 | 4 560 001 | 4 570 000 | 0.10 | 17 | *RPGRIP1L* |
| 11 | 18 460 001 | 18 470 000 | 0.07 | 204 | *SPIRE2* |
| 11 | 18 460 001 | 18 470 000 | 0.07 | 204 | *TCF25* |
| 11 | 18 470 001 | 18 480 000 | 0.09 | 181 | *TCF25* |
| 11 | 18 480 001 | 18 490 000 | 0.12 | 167 | *TCF25* |
| 11 | 18 480 001 | 18 490 000 | 0.12 | 167 | *MC1R* |
| 11 | 18 480 001 | 18 490 000 | 0.12 | 167 | *TUBB3* |
| 11 | 18 590 001 | 18 600 000 | 0.06 | 156 | *TERF2* |
| 11 | 18 590 001 | 18 600 000 | 0.06 | 156 | *CYB5B* |
| 11 | 18 600 001 | 18 610 000 | 0.07 | 130 | *CYB5B* |
| 11 | 18 610 001 | 18 620 000 | 0.08 | 174 | *NFAT5* |
| 12 | 220 001 | 230 000 | 0.07 | 60 | *POC1A* |
| 12 | 230 001 | 240 000 | 0.07 | 103 | *POC1A* |
| 12 | 250 001 | 260 000 | 0.08 | 72 | *POC1A* |
| 12 | 4 580 001 | 4 590 000 | 0.07 | 153 | *ATG7* |
| 12 | 7 910 001 | 7 920 000 | 0.07 | 48 | *CACNA2D3* |
| 12 | 8 800 001 | 8 810 000 | 0.06 | 154 | *APPL1* |
| 13 | 480 001 | 490 000 | 0.07 | 59 | *NRG2* |
| 13 | 490 001 | 500 000 | 0.07 | 53 | *NRG2* |
| 13 | 2 170 001 | 2 180 000 | 0.07 | 223 | *SIL1* |
| 13 | 17 000 001 | 17 010 000 | 0.07 | 128 | *NR3C1* |
| 13 | 17 010 001 | 17 020 000 | 0.07 | 131 | *NR3C1* |
| 14 | 3 020 001 | 3 030 000 | 0.06 | 211 | *MAD1L1* |
| 14 | 3 030 001 | 3 040 000 | 0.07 | 259 | *MAD1L1* |
| 14 | 3 620 001 | 3 630 000 | 0.06 | 112 | *SDK1* |
| 14 | 4 600 001 | 4 610 000 | 0.09 | 187 | *USP22* |
| 14 | 8 520 001 | 8 530 000 | 0.06 | 131 | *TMC5* |
| 14 | 8 640 001 | 8 650 000 | 0.06 | 243 | *LOC771638* |
| 14 | 8 640 001 | 8 650 000 | 0.06 | 243 | *SDR42E2* |
| 14 | 13 500 001 | 13 510 000 | 0.06 | 217 | *PRR35* |
| 14 | 13 500 001 | 13 510 000 | 0.06 | 217 | *NHLRC4* |
| 14 | 13 500 001 | 13 510 000 | 0.06 | 217 | *PIGQ* |
| 15 | 520 001 | 530 000 | 0.07 | 61 | *MAPK3* |
| 15 | 2 670 001 | 2 680 000 | 0.06 | 149 | *ULK1* |
| 15 | 5 010 001 | 5 020 000 | 0.06 | 144 | *KMT5A* |
| 15 | 5 010 001 | 5 020 000 | 0.06 | 144 | *SBNO1* |
| 15 | 8 590 001 | 8 600 000 | 0.07 | 214 | *BCR* |
| 15 | 10 030 001 | 10 040 000 | 0.08 | 475 | *POLE* |
| 15 | 10 030 001 | 10 040 000 | 0.08 | 475 | *CARD8* |
| 17 | 1 720 001 | 1 730 000 | 0.07 | 274 | *LOC427775* |
| 17 | 1 720 001 | 1 730 000 | 0.07 | 274 | *ZNF618* |
| 17 | 9 580 001 | 9 590 000 | 0.06 | 112 | *CRB2* |
| 18 | 6 820 001 | 6 830 000 | 0.07 | 78 | *NOG* |
| 19 | 1 100 001 | 1 110 000 | 0.09 | 236 | *RNF43* |
| 19 | 1 100 001 | 1 110 000 | 0.09 | 236 | *LOC112530078* |
| 19 | 1 110 001 | 1 120 000 | 0.09 | 315 | *LOC112530078* |
| 19 | 1 100 001 | 1 110 000 | 0.09 | 236 | *HSF5* |
| 19 | 1 110 001 | 1 120 000 | 0.09 | 315 | *HSF5* |
| 19 | 1 120 001 | 1 130 000 | 0.23 | 237 | *HSF5* |
| 19 | 1 130 001 | 1 140 000 | 0.15 | 187 | *HSF5* |
| 19 | 1 130 001 | 1 140 000 | 0.15 | 187 | *MTMR4* |
| 19 | 1 140 001 | 1 150 000 | 0.06 | 245 | *MTMR4* |
| 19 | 2 130 001 | 2 140 000 | 0.06 | 165 | *AUTS2* |
| 20 | 1 790 001 | 1 800 000 | 0.07 | 113 | *ITCH* |
| 20 | 10 960 001 | 10 970 000 | 0.07 | 170 | *NCOA5* |
| 20 | 10 960 001 | 10 970 000 | 0.07 | 170 | *LOC124417323* |
| 20 | 10 960 001 | 10 970 000 | 0.07 | 170 | *CD40* |
| 20 | 11 040 001 | 11 050 000 | 0.07 | 213 | *CDH22* |
| 20 | 11 100 001 | 11 110 000 | 0.10 | 223 | *DDX27* |
| 20 | 11 140 001 | 11 150 000 | 0.07 | 111 | *EDN3* |
| 20 | 11 180 001 | 11 190 000 | 0.06 | 180 | *ZNF831* |
| 20 | 11 190 001 | 11 200 000 | 0.09 | 192 | *ZNF831* |
| 20 | 11 200 001 | 11 210 000 | 0.07 | 208 | *ZNF831* |
| 20 | 11 210 001 | 11 220 000 | 0.10 | 245 | *PRELID3B* |
| 20 | 11 220 001 | 11 230 000 | 0.14 | 226 | *PRELID3B* |
| 20 | 11 210 001 | 11 220 000 | 0.10 | 245 | *LOC101747896* |
| 20 | 11 220 001 | 11 230 000 | 0.14 | 226 | *LOC101747896* |
| 20 | 11 220 001 | 11 230 000 | 0.14 | 226 | *ATP5E* |
| 20 | 11 220 001 | 11 230 000 | 0.14 | 226 | *TUBB1* |
| 20 | 11 230 001 | 11 240 000 | 0.09 | 214 | *TUBB1* |
| 20 | 11 230 001 | 11 240 000 | 0.09 | 214 | *CTSZ* |
| 20 | 11 240 001 | 11 250 000 | 0.07 | 171 | *CTSZ* |
| 20 | 11 240 001 | 11 250 000 | 0.07 | 171 | *NELFCD* |
| 20 | 11 250 001 | 11 260 000 | 0.07 | 219 | *NELFCD* |
| 20 | 11 250 001 | 11 260 000 | 0.07 | 219 | *GNAS* |
| 20 | 11 260 001 | 11 270 000 | 0.07 | 162 | *GNAS* |
| 20 | 12 330 001 | 12 340 000 | 0.07 | 225 | *RTFDC1* |
| 20 | 12 330 001 | 12 340 000 | 0.07 | 225 | *CASS4* |
| 20 | 12 860 001 | 12 870 000 | 0.07 | 68 | *LOC107048990* |
| 20 | 13 560 001 | 13 570 000 | 0.08 | 50 | *NFATC2* |
| 20 | 13 580 001 | 13 590 000 | 0.09 | 76 | *NFATC2* |
| 20 | 13 590 001 | 13 600 000 | 0.06 | 92 | *NFATC2* |
| 20 | 14 050 001 | 14 060 000 | 0.06 | 19 | *CEBPB* |
| 21 | 250 001 | 260 000 | 0.09 | 67 | *PARK7* |
| 21 | 250 001 | 260 000 | 0.09 | 67 | *TNFRSF9* |
| 21 | 260 001 | 270 000 | 0.08 | 75 | *TNFRSF9* |
| 21 | 330 001 | 340 000 | 0.09 | 145 | *CAMTA1* |
| 21 | 340 001 | 350 000 | 0.07 | 187 | *CAMTA1* |
| 23 | 6 010 001 | 6 020 000 | 0.07 | 19 | *MYOM3* |
| 23 | 6 100 001 | 6 110 000 | 0.07 | 17 | *GRHL3* |
| 24 | 6 160 001 | 6 170 000 | 0.12 | 141 | *PTS* |
| 24 | 6 160 001 | 6 170 000 | 0.12 | 141 | *BCO2* |
| 24 | 6 170 001 | 6 180 000 | 0.15 | 119 | *BCO2* |
| 24 | 6 180 001 | 6 190 000 | 0.16 | 100 | *BCO2* |
| 24 | 6 190 001 | 6 200 000 | 0.12 | 95 | *TEX12* |
| 24 | 6 190 001 | 6 200 000 | 0.12 | 95 | *IL18* |
| 24 | 6 220 001 | 6 230 000 | 0.06 | 108 | *DLAT* |
| 24 | 6 220 001 | 6 230 000 | 0.06 | 108 | *DIXDC1* |
| 24 | 6 230 001 | 6 240 000 | 0.06 | 73 | *DIXDC1* |
| 24 | 6 240 001 | 6 250 000 | 0.06 | 89 | *DIXDC1* |
| 24 | 6 250 001 | 6 260 000 | 0.07 | 112 | *DIXDC1* |
| 24 | 6 250 001 | 6 260 000 | 0.07 | 112 | *C11orf52* |
| 24 | 6 270 001 | 6 280 000 | 0.08 | 101 | *CRYAB* |
| 24 | 6 270 001 | 6 280 000 | 0.08 | 101 | *C11orf1* |
| 24 | 6 270 001 | 6 280 000 | 0.08 | 101 | *FDXACB1* |
| 24 | 6 280 001 | 6 290 000 | 0.07 | 123 | *FDXACB1* |
| 24 | 6 280 001 | 6 290 000 | 0.07 | 123 | *ALG9* |
| 24 | 6 290 001 | 6 300 000 | 0.07 | 130 | *ALG9* |
| 25 | 2 740 001 | 2 750 000 | 0.06 | 112 | *POGZ* |
| 27 | 1 420 001 | 1 430 000 | 0.09 | 307 | *ASIC2* |
| 27 | 4 650 001 | 4 660 000 | 0.06 | 118 | *HAP1* |
| 27 | 4 650 001 | 4 660 000 | 0.06 | 118 | *LOC112530405* |
| 27 | 4 650 001 | 4 660 000 | 0.06 | 118 | *JUP* |
| 28 | 4 900 001 | 4 910 000 | 0.08 | 133 | *LOC107055376* |
| 31 | 780 001 | 790 000 | 0.08 | 153 | *LOC107049379* |
| 34 | 1 210 001 | 1 220 000 | 0.07 | 27 | *ZNF740* |
| 34 | 1 210 001 | 1 220 000 | 0.07 | 27 | *LOC107049666* |
| 34 | 2 470 001 | 2 480 000 | 0.07 | 21 | *PIP4K2C* |

**Supplementary Table 6**

Genes overlapping genomic windows in the top 1% of GWAS signals for carnation-comb versus non-carnation-comb chickens.

| Chr | Site | -log10(*p*) | Gene |
| --- | --- | --- | --- |
| 19 | 1 116 839 | 172.79 | *HSF5* |
| 19 | 1 118 491 | 149.70 | *HSF5* |
| 19 | 1 118 100 | 145.03 | *HSF5* |
| 19 | 1 118 543 | 118.12 | *HSF5* |
| 19 | 1 119 114 | 111.46 | *HSF5* |
| 19 | 1 119 061 | 90.13 | *HSF5* |
| 19 | 1 119 063 | 90.13 | *HSF5* |
| 19 | 1 125 544 | 85.08 | *HSF5* |
| 19 | 1 117 869 | 65.13 | *HSF5* |
| 19 | 1 120 691 | 57.70 | *HSF5* |
| 19 | 1 119 366 | 57.55 | *HSF5* |
| 19 | 1 121 720 | 57.55 | *HSF5* |
| 19 | 1 120 697 | 56.66 | *HSF5* |
| 19 | 1 120 560 | 56.26 | *HSF5* |
| 19 | 1 120 655 | 55.92 | *HSF5* |
| 19 | 1 120 414 | 52.74 | *HSF5* |
| 19 | 1 119 858 | 52.54 | *HSF5* |
| 19 | 1 119 860 | 52.54 | *HSF5* |
| 19 | 1 120 012 | 52.32 | *HSF5* |
| 19 | 1 119 905 | 51.20 | *HSF5* |
| 19 | 1 119 876 | 51.14 | *HSF5* |
| 19 | 1 119 497 | 50.18 | *HSF5* |
| 19 | 1 120 671 | 50.07 | *HSF5* |
| 19 | 1 120 006 | 49.77 | *HSF5* |
| 19 | 1 121 539 | 48.83 | *HSF5* |
| 19 | 1 121 359 | 47.47 | *HSF5* |
| 19 | 1 120 719 | 47.15 | *HSF5* |
| 19 | 1 126 061 | 47.08 | *HSF5* |
| 19 | 1 121 553 | 47.04 | *HSF5* |
| 19 | 1 120 942 | 45.84 | *HSF5* |
| 19 | 1 132 250 | 45.15 | *HSF5* |
| 19 | 1 132 250 | 45.15 | *MTMR4* |
| 19 | 1 120 220 | 44.94 | *HSF5* |
| 19 | 1 120 225 | 44.94 | *HSF5* |
| 19 | 1 120 496 | 44.35 | *HSF5* |
| 19 | 1 122 287 | 44.13 | *HSF5* |
| 19 | 1 121 972 | 44.09 | *HSF5* |
| 19 | 1 120 921 | 43.73 | *HSF5* |
| 19 | 1 130 971 | 42.98 | *HSF5* |
| 19 | 1 130 971 | 42.98 | *MTMR4* |
| 19 | 1 123 055 | 42.82 | *HSF5* |
| 19 | 1 120 069 | 41.61 | *HSF5* |
| 19 | 1 120 088 | 41.61 | *HSF5* |
| 19 | 1 122 810 | 40.66 | *HSF5* |
| 19 | 1 122 813 | 40.66 | *HSF5* |
| 19 | 1 122 816 | 40.66 | *HSF5* |
| 19 | 1 122 817 | 40.66 | *HSF5* |
| 19 | 1 121 993 | 40.47 | *HSF5* |
| 19 | 1 122 833 | 40.01 | *HSF5* |
| 19 | 1 122 844 | 38.71 | *HSF5* |
| 19 | 1 129 758 | 38.22 | *HSF5* |
| 19 | 1 129 758 | 38.22 | *MTMR4* |
| 19 | 1 124 974 | 38.18 | *HSF5* |
| 19 | 1 120 431 | 37.44 | *HSF5* |
| 19 | 1 123 497 | 35.80 | *HSF5* |
| 19 | 1 132 178 | 33.78 | *HSF5* |
| 19 | 1 132 178 | 33.78 | *MTMR4* |
| 19 | 1 120 374 | 32.63 | *HSF5* |
| 19 | 1 123 611 | 31.54 | *HSF5* |
| 19 | 1 127 538 | 28.44 | *HSF5* |
| 19 | 1 126 994 | 21.27 | *HSF5* |
| 19 | 1 126 706 | 21.06 | *HSF5* |
| 1 | 120 815 830 | 18.15 | *RAI2* |
| 1 | 120 648 092 | 16.24 | *CDKL5* |
| 1 | 136 586 067 | 15.28 | *SH3RF3* |
| 27 | 1 416 561 | 15.23 | *ASIC2* |
| 11 | 18 472 456 | 14.93 | *TCF25* |
| 5 | 14 993 352 | 14.51 | *TSPAN4* |
| 5 | 15 005 614 | 14.51 | *TSPAN4* |
| 11 | 18 478 437 | 14.51 | *TCF25* |
| 11 | 18 478 437 | 14.51 | *MC1R* |
| 5 | 14 989 203 | 14.44 | *TSPAN4* |
| 5 | 15 040 419 | 14.40 | *TSPAN4* |
| 5 | 15 040 597 | 14.40 | *TSPAN4* |
| 5 | 14 888 307 | 14.34 | *TSPAN4* |
| 1 | 120 446 421 | 14.28 | *ADGRG2* |
| 8 | 7 546 346 | 14.24 | *NMNAT2* |
| 19 | 1 126 472 | 13.93 | *HSF5* |
| 19 | 1 136 108 | 13.87 | *MTMR4* |
| 19 | 1 119 335 | 13.82 | *HSF5* |
| 19 | 1 133 556 | 13.82 | *HSF5* |
| 19 | 1 133 556 | 13.82 | *MTMR4* |
| 1 | 131 195 515 | 13.74 | *GABRG3* |
| 4 | 11 120 563 | 13.67 | *EDNRB2* |
| 4 | 11 120 563 | 13.67 | *POLR1D* |
| 19 | 1 118 985 | 13.60 | *HSF5* |
| 1 | 173 796 795 | 13.43 | *STARD13* |
| 1 | 173 796 637 | 13.09 | *STARD13* |
| 19 | 1 127 389 | 13.05 | *HSF5* |
| 19 | 1 127 414 | 13.04 | *HSF5* |
| 1 | 131 195 383 | 12.92 | *GABRG3* |
| 1 | 131 262 569 | 12.84 | *GABRG3* |
| 19 | 1 119 960 | 12.81 | *HSF5* |
| 1 | 131 195 472 | 12.69 | *GABRG3* |
| 19 | 1 135 095 | 12.67 | *MTMR4* |
| 19 | 1 127 451 | 12.57 | *HSF5* |
| 1 | 131 195 510 | 12.28 | *GABRG3* |
| 1 | 131 195 499 | 12.22 | *GABRG3* |
| 19 | 1 129 274 | 12.06 | *HSF5* |
| 19 | 1 129 274 | 12.06 | *MTMR4* |
| 5 | 15 023 083 | 11.82 | *TSPAN4* |
| 6 | 2 788 841 | 11.78 | *GRID1* |
| 5 | 15 014 437 | 11.74 | *TSPAN4* |
| 1 | 120 563 059 | 11.73 | *PPEF1* |
| 1 | 21 228 289 | 11.72 | *GRM8* |
| 1 | 21 406 229 | 11.72 | *GRM8* |
| 6 | 2 767 159 | 11.61 | *GRID1* |
| 19 | 1 129 271 | 11.56 | *HSF5* |
| 19 | 1 129 271 | 11.56 | *MTMR4* |
| 11 | 18 486 202 | 11.50 | *MC1R* |
| 11 | 18 486 202 | 11.50 | *TUBB3* |
| 11 | 18 486 202 | 11.50 | *DEF8* |
| 19 | 1 137 066 | 11.47 | *MTMR4* |
| 4 | 88 291 396 | 11.46 | *LZTS3* |
| 11 | 18 464 493 | 11.44 | *SPIRE2* |
| 11 | 18 464 493 | 11.44 | *TCF25* |
| 1 | 8 241 840 | 11.38 | *GRM3* |
| 8 | 6 780 342 | 11.32 | *PAPPA2* |
| 8 | 6 730 872 | 11.29 | *BRINP2* |
| 8 | 6 730 872 | 11.29 | *ASTN1* |
| 4 | 88 287 828 | 11.28 | *LZTS3* |
| 1 | 47 793 588 | 11.28 | *NUP205* |
| 1 | 120 555 171 | 11.28 | *PPEF1* |
| 19 | 1 123 468 | 11.24 | *HSF5* |
| 17 | 5 622 638 | 11.23 | *URM1* |
| 11 | 18 481 914 | 11.17 | *TCF25* |
| 11 | 18 481 914 | 11.17 | *MC1R* |
| 11 | 18 481 914 | 11.17 | *TUBB3* |
| 1 | 120 650 239 | 11.16 | *CDKL5* |
| 11 | 18 478 079 | 11.08 | *TCF25* |
| 11 | 18 478 079 | 11.08 | *MC1R* |
| 5 | 15 007 517 | 11.07 | *TSPAN4* |
| 19 | 1 134 832 | 10.87 | *MTMR4* |
| 11 | 18 483 266 | 10.79 | *TCF25* |
| 11 | 18 483 266 | 10.79 | *MC1R* |
| 11 | 18 483 266 | 10.79 | *TUBB3* |
| 11 | 18 483 266 | 10.79 | *DEF8* |
| 11 | 18 451 833 | 10.77 | *FANCA* |
| 11 | 18 451 833 | 10.77 | *SPIRE2* |
| 19 | 1 126 214 | 10.77 | *HSF5* |
| 2 | 127 653 659 | 10.66 | *VPS13B* |
| 3 | 5 286 401 | 10.61 | *MACROD2* |
| 11 | 18 466 359 | 10.60 | *SPIRE2* |
| 11 | 18 466 359 | 10.60 | *TCF25* |
| 19 | 1 107 558 | 10.50 | *LOC112530078* |
| 19 | 1 107 558 | 10.50 | *HSF5* |
| 1 | 120 484 417 | 10.45 | *ADGRG2* |
| 27 | 3 090 414 | 10.39 | *MPP3* |
| 27 | 3 090 414 | 10.39 | *C27H17ORF105* |
| 27 | 3 090 414 | 10.39 | *RAB18L* |
| 1 | 131 796 291 | 10.36 | *ATP10A* |
| 27 | 1 416 597 | 10.33 | *ASIC2* |
| 1 | 120 648 372 | 10.27 | *CDKL5* |
| 1 | 173 796 602 | 10.26 | *STARD13* |
| 5 | 15 020 292 | 10.24 | *TSPAN4* |
| 10 | 2 694 731 | 10.19 | *SIN3A* |
| 2 | 5 627 985 | 10.17 | *SCN5A* |
| 1 | 145 375 094 | 10.13 | *MBNL2* |
| 2 | 5 627 995 | 10.12 | *SCN5A* |
| 11 | 18 475 170 | 9.99 | *TCF25* |
| 2 | 41 559 940 | 9.90 | *ATP2C1* |
| 8 | 2 369 957 | 9.87 | *DENND1B* |
| 8 | 2 368 423 | 9.86 | *DENND1B* |
| 1 | 131 195 107 | 9.82 | *GABRG3* |
| 1 | 15 091 345 | 9.81 | *LAMB1* |
| 2 | 131 084 127 | 9.81 | *RSPO2* |
| 1 | 120 650 280 | 9.74 | *CDKL5* |
| 1 | 131 200 302 | 9.72 | *GABRG3* |
| 20 | 11 097 632 | 9.69 | *DDX27* |
| 3 | 67 558 345 | 9.64 | *PDSS2* |
| 1 | 176 330 119 | 9.59 | *GPR12* |
| 21 | 329 430 | 9.55 | *CAMTA1* |
| 19 | 1 105 811 | 9.53 | *LOC112530078* |
| 19 | 1 105 811 | 9.53 | *HSF5* |
| 11 | 18 454 202 | 9.48 | *FANCA* |
| 11 | 18 454 202 | 9.48 | *SPIRE2* |
| 14 | 6 963 771 | 9.48 | *COG7* |
| 14 | 6 963 771 | 9.48 | *SCNN1B* |
| 4 | 15 444 820 | 9.47 | *TENM1* |
| 1 | 47 802 094 | 9.43 | *NUP205* |
| 19 | 1 105 764 | 9.42 | *LOC112530078* |
| 19 | 1 105 764 | 9.42 | *HSF5* |
| 1 | 131 195 423 | 9.41 | *GABRG3* |
| 11 | 18 472 761 | 9.39 | *TCF25* |
| 11 | 18 472 767 | 9.39 | *TCF25* |
| 11 | 18 472 774 | 9.39 | *TCF25* |
| 19 | 1 136 643 | 9.35 | *MTMR4* |
| 20 | 12 329 617 | 9.35 | *RTFDC1* |
| 20 | 12 329 617 | 9.35 | *CASS4* |
| 19 | 1 047 870 | 9.32 | *RNF43* |
| 1 | 131 195 197 | 9.32 | *GABRG3* |
| 1 | 130 082 062 | 9.29 | *PPP2R3B* |
| 11 | 18 469 829 | 9.28 | *TCF25* |
| 19 | 1 125 314 | 9.28 | *HSF5* |
| 11 | 18 458 126 | 9.27 | *FANCA* |
| 11 | 18 458 126 | 9.27 | *SPIRE2* |
| 11 | 18 458 126 | 9.27 | *TCF25* |
| 2 | 5 627 289 | 9.26 | *SCN5A* |
| 1 | 120 668 136 | 9.24 | *CDKL5* |
| 27 | 1 417 034 | 9.21 | *ASIC2* |
| 1 | 120 650 322 | 9.21 | *CDKL5* |
| 19 | 1 104 320 | 9.19 | *RNF43* |
| 19 | 1 104 320 | 9.19 | *LOC112530078* |
| 19 | 1 104 320 | 9.19 | *HSF5* |
| 5 | 15 017 462 | 9.19 | *TSPAN4* |
| 20 | 12 332 786 | 9.18 | *RTFDC1* |
| 20 | 12 332 786 | 9.18 | *CASS4* |
| 20 | 11 097 445 | 9.17 | *DDX27* |
| 14 | 6 956 217 | 9.16 | *COG7* |
| 14 | 6 956 282 | 9.16 | *COG7* |
| 11 | 18 481 830 | 9.15 | *TCF25* |
| 11 | 18 481 830 | 9.15 | *MC1R* |
| 11 | 18 481 830 | 9.15 | *TUBB3* |
| 3 | 32 018 449 | 9.13 | *CRIM1* |
| 1 | 173 785 676 | 9.12 | *STARD13* |
| 19 | 1 167 689 | 9.11 | *MTMR4* |
| 1 | 132 941 781 | 9.09 | *AFF3* |
| 2 | 5 628 508 | 9.07 | *SCN5A* |
| 19 | 1 106 183 | 9.06 | *LOC112530078* |
| 19 | 1 106 183 | 9.06 | *HSF5* |
| 19 | 1 106 836 | 9.05 | *LOC112530078* |
| 19 | 1 106 836 | 9.05 | *HSF5* |
| 19 | 1 106 838 | 9.05 | *LOC112530078* |
| 19 | 1 106 838 | 9.05 | *HSF5* |
| 1 | 120 221 772 | 9.03 | *SH3KBP1* |
| 3 | 11 748 549 | 9.02 | *PCSK2* |
| 1 | 20 266 506 | 9.02 | *IL17REL* |
| 6 | 28 681 351 | 9.02 | *ABLIM1* |
| 14 | 6 742 022 | 9.01 | *CACNG3* |
| 19 | 1 103 838 | 9.01 | *RNF43* |
| 19 | 1 103 838 | 9.01 | *LOC112530078* |
| 19 | 1 103 838 | 9.01 | *HSF5* |
| 19 | 1 103 843 | 9.01 | *RNF43* |
| 19 | 1 103 843 | 9.01 | *LOC112530078* |
| 19 | 1 103 843 | 9.01 | *HSF5* |
| 19 | 1 103 847 | 9.01 | *RNF43* |
| 19 | 1 103 847 | 9.01 | *LOC112530078* |
| 19 | 1 103 847 | 9.01 | *HSF5* |
| 20 | 12 329 672 | 9.01 | *RTFDC1* |
| 20 | 12 329 672 | 9.01 | *CASS4* |
| 7 | 30 140 658 | 9.00 | *ZRANB3* |
| 1 | 20 266 392 | 9.00 | *IL17REL* |
| 3 | 28 493 878 | 8.99 | *DAAM2* |
| 1 | 120 443 293 | 8.99 | *ADGRG2* |
| 19 | 1 135 285 | 8.99 | *MTMR4* |
| 1 | 166 021 670 | 8.98 | *VWA8* |
| 1 | 178 438 534 | 8.96 | *ZDHHC20* |
| 1 | 178 444 533 | 8.96 | *ZDHHC20* |
| 1 | 178 446 173 | 8.96 | *ZDHHC20* |
| 1 | 178 463 975 | 8.96 | *ZDHHC20* |
| 1 | 178 463 975 | 8.96 | *MRPL57* |
| 1 | 178 463 975 | 8.96 | *SKA3* |
| 3 | 11 748 708 | 8.96 | *PCSK2* |
| 5 | 7 619 562 | 8.93 | *PARVA* |
| 1 | 23 502 822 | 8.92 | *CPED1* |
| 1 | 137 036 420 | 8.91 | *RASA3* |
| 1 | 137 092 774 | 8.91 | *RASA3* |
| 1 | 137 097 639 | 8.91 | *RASA3* |
| 1 | 137 097 643 | 8.91 | *RASA3* |
| 1 | 137 099 990 | 8.91 | *RASA3* |
| 1 | 137 100 454 | 8.91 | *RASA3* |
| 1 | 137 262 896 | 8.91 | *TMEM255B* |
| 1 | 137 265 933 | 8.91 | *TMEM255B* |
| 1 | 137 265 934 | 8.91 | *TMEM255B* |
| 1 | 137 267 527 | 8.91 | *TMEM255B* |
| 19 | 1 120 267 | 8.91 | *HSF5* |
| 10 | 17 752 021 | 8.91 | *CLPX* |
| 1 | 128 831 914 | 8.91 | *DHRSX* |
| 3 | 8 428 359 | 8.91 | *LOC124416957* |
| 3 | 8 429 427 | 8.91 | *LOC124416957* |
| 3 | 76 600 694 | 8.90 | *SYNCRIP* |
| 3 | 28 500 266 | 8.90 | *DAAM2* |
| 1 | 178 439 767 | 8.90 | *ZDHHC20* |
| 12 | 18 036 595 | 8.90 | *CNTN4* |
| 1 | 131 192 692 | 8.88 | *GABRG3* |
| 1 | 137 274 199 | 8.88 | *TMEM255B* |
| 1 | 137 034 310 | 8.87 | *RASA3* |
| 1 | 8 250 874 | 8.86 | *GRM3* |
| 19 | 1 134 983 | 8.86 | *MTMR4* |
| 6 | 30 722 732 | 8.86 | *LOC121111175* |
| 6 | 30 737 464 | 8.86 | *LOC121111175* |
| 3 | 5 119 888 | 8.85 | *MACROD2* |
| 3 | 5 384 649 | 8.85 | *MACROD2* |
| 3 | 5 409 943 | 8.85 | *MACROD2* |
| 1 | 130 535 029 | 8.83 | *CYFIP1* |
| 3 | 76 617 211 | 8.81 | *SNX14* |
| 1 | 137 104 637 | 8.81 | *RASA3* |
| 19 | 1 104 323 | 8.81 | *RNF43* |
| 19 | 1 104 323 | 8.81 | *LOC112530078* |
| 19 | 1 104 323 | 8.81 | *HSF5* |
| 1 | 17 673 457 | 8.80 | *FAM19A5* |
| 19 | 1 140 784 | 8.79 | *MTMR4* |
| 8 | 6 902 921 | 8.78 | *RFWD2* |
| 2 | 43 971 129 | 8.78 | *MOBP* |
| 2 | 43 974 847 | 8.78 | *MOBP* |
| 19 | 1 105 932 | 8.78 | *LOC112530078* |
| 19 | 1 105 932 | 8.78 | *HSF5* |
| 8 | 7 553 750 | 8.77 | *NMNAT2* |
| 14 | 6 493 055 | 8.77 | *MLST8* |
| 14 | 6 493 055 | 8.77 | *BRICD5* |
| 14 | 6 493 055 | 8.77 | *PGP* |
| 14 | 6 493 055 | 8.77 | *E4F1* |
| 11 | 17 398 776 | 8.77 | *KIAA0513* |
| 1 | 131 191 151 | 8.75 | *GABRG3* |
| 1 | 20 397 684 | 8.75 | *LOC429348* |
| 1 | 20 397 798 | 8.75 | *LOC429348* |
| 1 | 20 399 495 | 8.75 | *LOC429348* |
| 1 | 59 461 708 | 8.75 | *ETFBKMT* |
| 10 | 13 313 467 | 8.75 | *NTRK3* |
| 3 | 8 137 935 | 8.74 | *LCLAT1* |
| 19 | 1 130 469 | 8.74 | *HSF5* |
| 19 | 1 130 469 | 8.74 | *MTMR4* |
| 1 | 17 673 455 | 8.74 | *FAM19A5* |
| 3 | 10 183 218 | 8.73 | *ACTR2* |
| 6 | 30 999 907 | 8.73 | *RGS10* |
| 13 | 13 466 840 | 8.72 | *CNOT6* |
| 13 | 13 467 836 | 8.72 | *CNOT6* |
| 13 | 13 470 261 | 8.72 | *CNOT6* |
| 13 | 13 470 292 | 8.72 | *CNOT6* |
| 13 | 13 470 879 | 8.72 | *CNOT6* |
| 13 | 13 471 047 | 8.72 | *CNOT6* |
| 13 | 13 471 047 | 8.72 | *GFPT2* |
| 6 | 4 286 123 | 8.72 | *NRG3* |
| 3 | 29 873 926 | 8.72 | *RNF8* |
| 1 | 8 350 031 | 8.72 | *GRM3* |
| 1 | 9 804 226 | 8.72 | *SEMA3E* |
| 1 | 131 187 276 | 8.72 | *GABRG3* |
| 1 | 131 187 277 | 8.72 | *GABRG3* |
| 14 | 6 493 075 | 8.72 | *MLST8* |
| 14 | 6 493 075 | 8.72 | *BRICD5* |
| 14 | 6 493 075 | 8.72 | *PGP* |
| 14 | 6 493 075 | 8.72 | *E4F1* |
| 19 | 1 181 626 | 8.71 | *MTMR4* |
| 19 | 1 181 626 | 8.71 | *CASP1* |
| 19 | 1 181 626 | 8.71 | *ABHD11* |
| 3 | 28 507 313 | 8.69 | *DAAM2* |
| 3 | 78 646 666 | 8.69 | *LOC121110231* |
| 3 | 78 646 666 | 8.69 | *LOC121110233* |
| 34 | 973 924 | 8.68 | *ATF7* |
| 19 | 1 138 175 | 8.65 | *MTMR4* |
| 19 | 1 123 804 | 8.65 | *HSF5* |
| 11 | 18 471 536 | 8.63 | *TCF25* |
| 2 | 29 654 044 | 8.62 | *TWISTNB* |
| 2 | 29 895 723 | 8.62 | *ITGB8* |
| 2 | 29 896 033 | 8.62 | *ITGB8* |
| 2 | 29 897 960 | 8.62 | *ITGB8* |
| 2 | 32 287 195 | 8.62 | *LOC121110067* |
| 2 | 32 287 198 | 8.62 | *LOC121110067* |
| 1 | 175 063 953 | 8.62 | *KATNAL1* |
| 21 | 3 945 151 | 8.62 | *LOC124417345* |
| 19 | 1 103 900 | 8.61 | *RNF43* |
| 19 | 1 103 900 | 8.61 | *LOC112530078* |
| 19 | 1 103 900 | 8.61 | *HSF5* |
| 28 | 1 406 972 | 8.60 | *TJP3* |
| 2 | 24 751 679 | 8.59 | *UMAD1* |
| 15 | 4 347 367 | 8.59 | *TMEM132B* |
| 20 | 11 135 186 | 8.58 | *EDN3* |
| 11 | 18 479 508 | 8.58 | *TCF25* |
| 11 | 18 479 508 | 8.58 | *MC1R* |
| 2 | 41 816 902 | 8.58 | *CPNE4* |
| 2 | 41 694 975 | 8.58 | *NEK11* |
| 2 | 41 715 649 | 8.58 | *NEK11* |
| 2 | 42 094 697 | 8.58 | *LOC121109843* |
| 2 | 42 233 491 | 8.58 | *ACAD11* |
| 2 | 42 233 491 | 8.58 | *UBA5* |
| 2 | 42 526 721 | 8.58 | *BFSP2* |
| 2 | 42 103 678 | 8.57 | *LOC121109843* |
| 2 | 42 266 676 | 8.57 | *NPHP3* |
| 2 | 29 466 985 | 8.57 | *TWIST1* |
| 1 | 49 723 139 | 8.56 | *ZC3H7B* |
| 1 | 49 741 106 | 8.56 | *ZC3H7B* |
| 19 | 10 259 576 | 8.56 | *SLC13A5* |
| 19 | 9 324 821 | 8.56 | *MSI2* |
| 12 | 11 205 764 | 8.56 | *GRIP2* |
| 2 | 17 479 873 | 8.56 | *PIP4K2A* |
| 8 | 6 073 782 | 8.54 | *TOR1AIP1* |
| 8 | 6 073 782 | 8.54 | *TOR1AIP2* |
| 8 | 6 307 669 | 8.54 | *RALGPS2* |
| 8 | 6 358 638 | 8.54 | *RALGPS2* |
| 8 | 6 375 018 | 8.54 | *RALGPS2* |
| 8 | 6 485 826 | 8.54 | *SYNGAP1* |
| 8 | 6 527 005 | 8.54 | *SYNGAP1* |
| 8 | 6 534 453 | 8.54 | *SYNGAP1* |
| 8 | 6 549 126 | 8.54 | *SYNGAP1* |
| 2 | 64 431 423 | 8.53 | *RREB1* |
| 1 | 188 084 209 | 8.53 | *GRM5* |
| 2 | 128 926 365 | 8.53 | *BAALC* |
| 19 | 1 107 345 | 8.53 | *LOC112530078* |
| 19 | 1 107 345 | 8.53 | *HSF5* |
| 2 | 127 945 727 | 8.53 | *RGS22* |
| 1 | 121 224 306 | 8.53 | *NHS* |
| 1 | 121 339 300 | 8.53 | *REPS2* |
| 1 | 172 189 597 | 8.53 | *SUPT20H* |
| 1 | 172 189 597 | 8.53 | *EXOSC8* |
| 4 | 47 641 306 | 8.52 | *ADGRL3* |
| 8 | 6 348 882 | 8.52 | *RALGPS2* |
| 8 | 6 348 882 | 8.52 | *ANGPTL1* |
| 1 | 121 339 293 | 8.51 | *REPS2* |
| 8 | 6 549 169 | 8.51 | *SYNGAP1* |
| 1 | 120 370 755 | 8.51 | *MAP3K15* |
| 1 | 120 370 755 | 8.51 | *PDHA2* |
| 1 | 120 370 762 | 8.51 | *MAP3K15* |
| 1 | 120 370 762 | 8.51 | *PDHA2* |
| 8 | 6 349 516 | 8.51 | *RALGPS2* |
| 8 | 6 349 516 | 8.51 | *ANGPTL1* |
| 1 | 173 796 219 | 8.50 | *STARD13* |
| 3 | 105 101 642 | 8.50 | *OTOF* |
| 8 | 6 349 392 | 8.50 | *RALGPS2* |
| 8 | 6 349 392 | 8.50 | *ANGPTL1* |
| 3 | 30 068 088 | 8.49 | *MRPL14* |
| 19 | 1 136 153 | 8.47 | *MTMR4* |
| 1 | 123 846 616 | 8.47 | *FRMPD4* |
| 19 | 1 104 962 | 8.47 | *RNF43* |
| 19 | 1 104 962 | 8.47 | *LOC112530078* |
| 19 | 1 104 962 | 8.47 | *HSF5* |
| 28 | 4 279 527 | 8.47 | *CRTC1* |
| 28 | 4 279 528 | 8.47 | *CRTC1* |
| 4 | 12 954 725 | 8.46 | *AMOT* |
| 4 | 12 957 614 | 8.46 | *AMOT* |
| 4 | 12 961 176 | 8.46 | *AMOT* |
| 4 | 12 961 362 | 8.46 | *AMOT* |
| 2 | 22 364 452 | 8.46 | *AKAP9* |
| 4 | 314 175 | 8.46 | *LOC100859173* |
| 11 | 18 474 642 | 8.46 | *TCF25* |
| 11 | 18 474 643 | 8.46 | *TCF25* |
| 4 | 46 057 934 | 8.44 | *LOC422609* |
| 4 | 46 057 951 | 8.44 | *LOC422609* |
| 1 | 120 666 405 | 8.43 | *CDKL5* |
| 1 | 39 842 143 | 8.43 | *RPS16* |
| 4 | 59 369 369 | 8.43 | *TSPAN17* |
| 2 | 17 006 918 | 8.41 | *KIAA1217* |
| 4 | 59 369 303 | 8.41 | *TSPAN17* |
| 5 | 15 015 956 | 8.40 | *TSPAN4* |
| 2 | 32 331 467 | 8.40 | *SKAP2* |
| 2 | 32 392 740 | 8.40 | *SKAP2* |
| 2 | 58 924 826 | 8.40 | *CDKAL1* |
| 2 | 131 096 379 | 8.39 | *RSPO2* |
| 19 | 1 118 197 | 8.39 | *HSF5* |
| 19 | 1 107 545 | 8.37 | *LOC112530078* |
| 19 | 1 107 545 | 8.37 | *HSF5* |
| 27 | 1 358 996 | 8.36 | *SMARCD2* |
| 2 | 7 983 775 | 8.36 | *RBM33* |
| 1 | 105 440 810 | 8.34 | *GART-B* |
| 1 | 105 440 810 | 8.34 | *GART* |
| 1 | 76 843 772 | 8.33 | *VAMP1* |
| 1 | 76 843 772 | 8.33 | *SCNN1A* |
| 3 | 65 694 421 | 8.33 | *FYN* |
| 3 | 65 699 165 | 8.33 | *FYN* |
| 3 | 65 709 359 | 8.33 | *FYN* |
| 3 | 65 709 359 | 8.33 | *LOC121110407* |
| 3 | 65 709 359 | 8.33 | *TRAF3IP2* |
| 3 | 65 722 821 | 8.33 | *LOC121110407* |
| 3 | 65 722 821 | 8.33 | *TRAF3IP2* |
| 1 | 173 787 382 | 8.33 | *STARD13* |
| 3 | 76 054 861 | 8.33 | *AKIRIN2* |
| 3 | 76 054 867 | 8.33 | *AKIRIN2* |
| 3 | 76 055 291 | 8.33 | *AKIRIN2* |
| 14 | 6 857 242 | 8.32 | *TNRC6A* |
| 20 | 12 332 901 | 8.31 | *CASS4* |
| 19 | 1 105 748 | 8.31 | *LOC112530078* |
| 19 | 1 105 748 | 8.31 | *HSF5* |
| 3 | 65 719 640 | 8.31 | *LOC121110407* |
| 3 | 65 719 640 | 8.31 | *TRAF3IP2* |
| 2 | 146 214 208 | 8.30 | *TSNARE1* |
| 3 | 65 693 478 | 8.30 | *FYN* |
| 18 | 3 314 355 | 8.27 | *COX10* |
| 10 | 15 033 224 | 8.27 | *MCTP2* |
| 27 | 1 624 820 | 8.26 | *ASIC2* |
| 8 | 21 530 859 | 8.26 | *ATPAF1* |
| 19 | 1 170 264 | 8.24 | *MTMR4* |
| 11 | 18 454 512 | 8.24 | *FANCA* |
| 11 | 18 454 512 | 8.24 | *SPIRE2* |
| 2 | 144 004 234 | 8.23 | *KCNK9* |
| 20 | 11 219 733 | 8.23 | *PRELID3B* |
| 20 | 11 219 733 | 8.23 | *LOC101747896* |
| 20 | 11 219 733 | 8.23 | *ATP5E* |
| 2 | 130 033 545 | 8.22 | *ZFPM2* |
| 1 | 131 060 301 | 8.22 | *GABRG3* |
| 9 | 23 211 216 | 8.22 | *MED12L* |
| 3 | 63 567 405 | 8.21 | *DSE* |
| 1 | 173 785 276 | 8.20 | *STARD13* |
| 28 | 1 404 343 | 8.17 | *APBA3* |
| 28 | 1 404 343 | 8.17 | *TJP3* |
| 19 | 5 953 577 | 8.16 | *RTN4RL1* |
| 20 | 11 222 298 | 8.16 | *LOC101747896* |
| 20 | 11 222 298 | 8.16 | *ATP5E* |
| 20 | 11 222 298 | 8.16 | *TUBB1* |
| 11 | 18 475 421 | 8.16 | *TCF25* |
| 20 | 11 180 520 | 8.15 | *ZNF831* |
| 5 | 16 550 126 | 8.15 | *ZDHHC5* |
| 5 | 16 550 126 | 8.15 | *CLP1* |
| 5 | 16 550 126 | 8.15 | *YPEL4* |
| 5 | 16 550 126 | 8.15 | *SERPING1* |
| 23 | 421 037 | 8.15 | *ARID1A* |
| 1 | 2 895 016 | 8.14 | *PLXNA4* |
| 28 | 1 404 508 | 8.13 | *APBA3* |
| 28 | 1 404 508 | 8.13 | *TJP3* |
| 14 | 6 401 830 | 8.11 | *RAB26* |
| 14 | 6 413 603 | 8.11 | *RAB26* |
| 14 | 6 413 603 | 8.11 | *TRAF7* |
| 2 | 42 124 852 | 8.10 | *ACPP* |
| 28 | 1 413 135 | 8.10 | *TJP3* |
| 28 | 1 413 135 | 8.10 | *PIP5K1C* |
| 8 | 21 532 654 | 8.10 | *ATPAF1* |
| 1 | 131 187 379 | 8.10 | *GABRG3* |
| 10 | 2 611 894 | 8.09 | *LOC124417129* |
| 10 | 2 611 894 | 8.09 | *STRA6* |
| 1 | 2 938 008 | 8.09 | *PLXNA4* |
| 2 | 146 204 739 | 8.09 | *TSNARE1* |
| 9 | 23 230 356 | 8.08 | *MED12L* |
| 9 | 23 230 356 | 8.08 | *P2RY12* |
| 9 | 23 230 356 | 8.08 | *P2RY13* |
| 6 | 30 694 609 | 8.06 | *LOC121111175* |
| 1 | 50 810 030 | 8.05 | *CBX6* |
| 1 | 50 980 338 | 8.05 | *CSNK1E* |
| 1 | 1 722 243 | 8.05 | *NET1* |
| 11 | 18 479 481 | 8.05 | *TCF25* |
| 11 | 18 479 481 | 8.05 | *MC1R* |
| 1 | 120 652 707 | 8.05 | *CDKL5* |
| 10 | 2 577 319 | 8.04 | *SEMA7A* |
| 10 | 2 577 319 | 8.04 | *CYP11A1* |
| 2 | 143 956 808 | 8.03 | *KCNK9* |
| 20 | 12 332 979 | 8.02 | *CASS4* |
| 2 | 128 502 102 | 8.01 | *NCALD* |
| 8 | 3 998 680 | 8.00 | *CRIP2* |
| 2 | 64 501 270 | 8.00 | *RREB1* |
| 1 | 120 627 146 | 8.00 | *CDKL5* |
| 1 | 83 568 591 | 7.99 | *BOC* |
| 16 | 2 007 704 | 7.99 | *LOC121106913* |
| 16 | 2 007 704 | 7.99 | *BG8* |
| 2 | 61 436 323 | 7.98 | *LOC121106455* |
| 11 | 18 452 510 | 7.98 | *FANCA* |
| 11 | 18 452 510 | 7.98 | *SPIRE2* |
| 11 | 6 220 054 | 7.97 | *ADCY7* |
| 4 | 68 419 953 | 7.97 | *RBM47* |
| 3 | 106 644 839 | 7.97 | *PINX1* |
| 1 | 120 235 701 | 7.97 | *SH3KBP1* |
| 1 | 120 128 456 | 7.97 | *SH3KBP1* |
| 1 | 4 542 889 | 7.97 | *SFMBT2* |
| 19 | 1 129 713 | 7.95 | *HSF5* |
| 19 | 1 129 713 | 7.95 | *MTMR4* |
| 20 | 12 332 066 | 7.94 | *RTFDC1* |
| 20 | 12 332 066 | 7.94 | *CASS4* |
| 3 | 28 488 591 | 7.94 | *DAAM2* |
| 11 | 18 470 511 | 7.94 | *TCF25* |
| 1 | 131 049 046 | 7.94 | *GABRG3* |
| 34 | 1 196 061 | 7.94 | *ESPL1* |
| 34 | 1 196 061 | 7.94 | *CSAD* |
| 19 | 1 105 734 | 7.93 | *LOC112530078* |
| 19 | 1 105 734 | 7.93 | *HSF5* |
| 5 | 15 004 152 | 7.93 | *TSPAN4* |
| 1 | 131 262 632 | 7.90 | *GABRG3* |
| 2 | 42 273 576 | 7.89 | *NPHP3* |
| 3 | 30 068 110 | 7.89 | *MRPL14* |
| 8 | 28 109 668 | 7.88 | *PDE4B* |
| 27 | 1 092 038 | 7.88 | *MYO1D* |
| 3 | 31 998 682 | 7.87 | *CRIM1* |
| 19 | 1 163 987 | 7.86 | *MTMR4* |
| 3 | 29 887 167 | 7.86 | *RNF8* |
| 3 | 29 887 167 | 7.86 | *AARS2* |
| 1 | 120 457 119 | 7.86 | *ADGRG2* |
| 3 | 29 361 805 | 7.86 | *ZFAND3* |
| 3 | 77 370 431 | 7.85 | *SNAP91* |
| 19 | 1 126 296 | 7.83 | *HSF5* |
| 19 | 1 123 690 | 7.82 | *HSF5* |
| 2 | 42 760 864 | 7.82 | *SACM1L* |
| 2 | 42 760 864 | 7.82 | *LIMD1* |
| 3 | 30 668 767 | 7.82 | *DLK2* |
| 3 | 30 668 767 | 7.82 | *ABCC10* |
| 5 | 13 920 635 | 7.82 | *MOB2* |
| 24 | 1 597 227 | 7.81 | *NFRKB* |
| 19 | 1 531 918 | 7.80 | *CALN1* |
| 9 | 13 414 824 | 7.80 | *OSTN* |
| 15 | 9 720 248 | 7.80 | *CCDC60* |
| 15 | 9 720 248 | 7.80 | *HSPB8* |
| 15 | 9 720 248 | 7.80 | *LOC124416952* |
| 4 | 68 506 770 | 7.78 | *RHOH* |
| 8 | 28 114 485 | 7.77 | *PDE4B* |
| 3 | 76 231 540 | 7.77 | *ZNF292* |
| 17 | 8 774 158 | 7.76 | *UBAC1* |
| 1 | 173 791 148 | 7.75 | *STARD13* |
| 1 | 131 050 865 | 7.74 | *GABRG3* |
| 3 | 106 645 261 | 7.74 | *PINX1* |
| 4 | 15 444 272 | 7.73 | *TENM1* |
| 10 | 13 298 793 | 7.73 | *NTRK3* |
| 1 | 131 187 627 | 7.72 | *GABRG3* |
| 18 | 1 562 920 | 7.71 | *MYOCD* |
| 1 | 104 563 968 | 7.70 | *TIAM1* |
| 3 | 34 167 421 | 7.70 | *KIF26B* |
| 1 | 120 364 471 | 7.70 | *MAP3K15* |
| 1 | 120 364 471 | 7.70 | *PDHA2* |
| 19 | 1 130 462 | 7.69 | *HSF5* |
| 19 | 1 130 462 | 7.69 | *MTMR4* |
| 11 | 18 477 833 | 7.69 | *TCF25* |
| 11 | 18 477 833 | 7.69 | *MC1R* |
| 18 | 3 305 457 | 7.69 | *COX10* |
| 1 | 16 033 616 | 7.68 | *CELSR1* |
| 3 | 5 232 865 | 7.68 | *MACROD2* |
| 5 | 21 709 543 | 7.67 | *TSPAN18* |
| 5 | 21 709 543 | 7.67 | *TP53I11* |
| 5 | 55 883 324 | 7.66 | *SOCS4* |
| 3 | 49 353 232 | 7.66 | *RGS17* |
| 1 | 179 984 154 | 7.64 | *DDX10* |
| 12 | 12 985 957 | 7.64 | *PTPRG* |
| 14 | 6 397 821 | 7.63 | *RAB26* |
| 6 | 4 458 093 | 7.63 | *NRG3* |
| 1 | 88 996 243 | 7.62 | *CGGBP1* |
| 1 | 88 996 243 | 7.62 | *ZNF654* |
| 1 | 89 002 393 | 7.62 | *ZNF654* |
| 1 | 89 013 413 | 7.62 | *ZNF654* |
| 4 | 34 640 160 | 7.62 | *HERC3* |
| 1 | 131 060 191 | 7.62 | *GABRG3* |
| 9 | 15 302 787 | 7.62 | *CHRNG* |
| 9 | 15 302 787 | 7.62 | *EIF4E2* |
| 8 | 28 108 232 | 7.61 | *PDE4B* |
| 3 | 9 130 987 | 7.61 | *PPP1R21* |
| 3 | 9 130 990 | 7.61 | *PPP1R21* |
| 3 | 9 130 994 | 7.61 | *PPP1R21* |
| 2 | 41 640 464 | 7.61 | *NEK11* |
| 27 | 1 419 141 | 7.61 | *ASIC2* |
| 28 | 3 965 616 | 7.61 | *SUGP1* |
| 1 | 120 211 522 | 7.61 | *SH3KBP1* |
| 1 | 179 988 143 | 7.60 | *DDX10* |
| 1 | 179 988 165 | 7.60 | *DDX10* |
| 1 | 179 988 167 | 7.60 | *DDX10* |
| 1 | 179 988 170 | 7.60 | *DDX10* |
| 1 | 179 988 172 | 7.60 | *DDX10* |
| 8 | 28 113 703 | 7.59 | *PDE4B* |
| 1 | 89 010 743 | 7.58 | *ZNF654* |
| 14 | 3 621 304 | 7.58 | *SDK1* |
| 28 | 578 166 | 7.57 | *LOC107055329* |
| 10 | 4 035 670 | 7.57 | *HERC1* |
| 1 | 17 627 565 | 7.57 | *FAM19A5* |
| 1 | 129 830 915 | 7.57 | *SHOX* |
| 1 | 130 062 770 | 7.57 | *PPP2R3B* |
| 1 | 130 138 965 | 7.57 | *STAG3* |
| 1 | 130 193 715 | 7.57 | *PLCXD1* |
| 1 | 130 274 992 | 7.57 | *JADE3* |
| 1 | 173 796 245 | 7.57 | *STARD13* |
| 3 | 31 999 388 | 7.56 | *CRIM1* |
| 2 | 143 982 444 | 7.56 | *KCNK9* |
| 1 | 131 187 317 | 7.55 | *GABRG3* |
| 10 | 17 597 400 | 7.55 | *CHSY1* |
| 19 | 1 118 425 | 7.54 | *HSF5* |
| 2 | 14 048 143 | 7.54 | *EPC1* |
| 2 | 14 048 263 | 7.54 | *EPC1* |
| 2 | 62 487 755 | 7.53 | *HIVEP1* |
| 17 | 1 634 910 | 7.53 | *NDOR1* |
| 17 | 1 634 910 | 7.53 | *LOC124417284* |
| 20 | 11 210 608 | 7.53 | *PRELID3B* |
| 20 | 11 210 608 | 7.53 | *LOC101747896* |
| 2 | 127 640 647 | 7.52 | *VPS13B* |
| 11 | 18 486 338 | 7.52 | *MC1R* |
| 11 | 18 486 338 | 7.52 | *TUBB3* |
| 11 | 18 486 338 | 7.52 | *DEF8* |
| 19 | 1 118 754 | 7.51 | *HSF5* |
| 11 | 18 483 113 | 7.51 | *TCF25* |
| 11 | 18 483 113 | 7.51 | *MC1R* |
| 11 | 18 483 113 | 7.51 | *TUBB3* |
| 4 | 34 588 246 | 7.51 | *CCNG2* |
| 1 | 131 050 743 | 7.51 | *GABRG3* |
| 2 | 5 628 348 | 7.49 | *SCN5A* |
| 2 | 29 934 817 | 7.49 | *ITGB8* |
| 7 | 30 139 806 | 7.49 | *ZRANB3* |
| 3 | 80 502 237 | 7.48 | *COL12A1* |
| 20 | 12 727 749 | 7.48 | *BCAS1* |
| 8 | 21 499 245 | 7.48 | *MKNK1* |
| 8 | 6 971 115 | 7.48 | *RFWD2* |
| 4 | 67 761 617 | 7.48 | *SHISA3* |
| 19 | 9 289 477 | 7.47 | *MSI2* |
| 1 | 173 816 138 | 7.47 | *STARD13* |
| 1 | 136 591 567 | 7.47 | *SH3RF3* |
| 8 | 7 195 651 | 7.46 | *RABGAP1L* |
| 6 | 27 841 701 | 7.45 | *TCF7L2* |
| 20 | 11 219 311 | 7.44 | *PRELID3B* |
| 20 | 11 219 311 | 7.44 | *LOC101747896* |
| 20 | 11 219 311 | 7.44 | *ATP5E* |
| 3 | 7 366 191 | 7.44 | *NRXN1* |
| 17 | 5 734 083 | 7.44 | *DNM1* |
| 3 | 77 520 372 | 7.44 | *ME1* |
| 3 | 4 456 681 | 7.44 | *KLHDC3* |
| 1 | 21 005 214 | 7.43 | *ZNF800* |
| 3 | 65 714 570 | 7.42 | *LOC121110407* |
| 3 | 65 714 570 | 7.42 | *TRAF3IP2* |
| 15 | 5 468 781 | 7.41 | *ANAPC5* |
| 28 | 996 740 | 7.40 | *SPPL2C* |
| 1 | 21 007 214 | 7.40 | *ZNF800* |
| 2 | 42 103 859 | 7.40 | *LOC121109843* |
| 9 | 23 206 089 | 7.39 | *MED12L* |
| 2 | 131 064 888 | 7.38 | *RSPO2* |
| 11 | 18 455 450 | 7.38 | *FANCA* |
| 11 | 18 455 450 | 7.38 | *SPIRE2* |
| 3 | 28 507 485 | 7.38 | *DAAM2* |
| 13 | 3 579 620 | 7.37 | *FOXI1* |
| 3 | 28 507 377 | 7.37 | *DAAM2* |
| 4 | 68 408 175 | 7.36 | *RBM47* |
| 1 | 14 181 816 | 7.36 | *PUS7* |
| 4 | 34 644 816 | 7.34 | *HERC3* |
| 1 | 170 951 120 | 7.34 | *LHFPL1* |
| 2 | 120 762 988 | 7.34 | *ZNF704* |
| 2 | 120 762 988 | 7.34 | *LOC124417753* |
| 27 | 1 362 035 | 7.34 | *SMARCD2* |
| 1 | 189 556 373 | 7.34 | *DLG2* |
| 4 | 34 636 935 | 7.33 | *HERC3* |
| 20 | 10 841 852 | 7.33 | *LOC771994* |
| 20 | 10 841 852 | 7.33 | *DNTTIP1* |
| 20 | 10 841 852 | 7.33 | *UBE2U* |
| 20 | 10 841 852 | 7.33 | *LOC107054848* |
| 1 | 178 460 731 | 7.33 | *ZDHHC20* |
| 17 | 1 455 519 | 7.33 | *EXD3* |
| 3 | 5 291 946 | 7.33 | *MACROD2* |
| 14 | 2 274 616 | 7.30 | *CYP2W2* |
| 14 | 2 274 616 | 7.30 | *C7orf50* |
| 14 | 2 274 933 | 7.30 | *CYP2W2* |
| 14 | 2 274 933 | 7.30 | *C7orf50* |
| 14 | 2 287 621 | 7.30 | *C7orf50* |
| 1 | 130 574 115 | 7.29 | *NIPA1* |
| 3 | 104 979 490 | 7.29 | *CCDC25* |
| 3 | 104 979 490 | 7.29 | *SCARA3* |
| 1 | 131 204 527 | 7.28 | *GABRG3* |
| 19 | 1 106 088 | 7.28 | *LOC112530078* |
| 19 | 1 106 088 | 7.28 | *HSF5* |
| 1 | 15 078 417 | 7.27 | *LAMB1* |
| 1 | 15 556 987 | 7.27 | *KIF21A* |
| 1 | 15 566 511 | 7.27 | *KIF21A* |
| 1 | 16 137 110 | 7.27 | *GRAMD4* |
| 1 | 16 144 116 | 7.27 | *GRAMD4* |
| 1 | 178 463 860 | 7.27 | *ZDHHC20* |
| 1 | 178 463 860 | 7.27 | *MRPL57* |
| 1 | 178 465 382 | 7.27 | *ZDHHC20* |
| 1 | 178 465 382 | 7.27 | *MRPL57* |
| 1 | 178 465 382 | 7.27 | *SKA3* |
| 1 | 178 465 614 | 7.27 | *ZDHHC20* |
| 1 | 178 465 614 | 7.27 | *MRPL57* |
| 1 | 178 465 614 | 7.27 | *SKA3* |
| 13 | 13 462 867 | 7.27 | *CNOT6* |
| 3 | 3 419 593 | 7.27 | *ZC3H8* |
| 26 | 3 357 206 | 7.26 | *KCND3* |
| 2 | 135 406 401 | 7.26 | *COLEC10* |
| 1 | 16 136 218 | 7.26 | *GRAMD4* |
| 1 | 16 129 951 | 7.26 | *GRAMD4* |
| 1 | 16 137 439 | 7.25 | *GRAMD4* |
| 15 | 11 005 414 | 7.25 | *ASCC2* |
| 2 | 3 441 348 | 7.23 | *MYL3* |
| 4 | 39 024 071 | 7.23 | *ACSL1* |
| 1 | 121 149 264 | 7.23 | *NHS* |
| 1 | 116 495 451 | 7.22 | *IL1RAPL1* |
| 1 | 116 506 438 | 7.22 | *IL1RAPL1* |
| 1 | 116 506 494 | 7.22 | *IL1RAPL1* |
| 1 | 116 524 374 | 7.22 | *IL1RAPL1* |
| 1 | 116 524 932 | 7.22 | *IL1RAPL1* |
| 19 | 1 093 756 | 7.22 | *RNF43* |
| 1 | 116 498 887 | 7.22 | *IL1RAPL1* |
| 3 | 28 506 769 | 7.21 | *DAAM2* |
| 1 | 116 496 347 | 7.21 | *IL1RAPL1* |
| 5 | 55 883 212 | 7.21 | *SOCS4* |
| 1 | 20 226 315 | 7.20 | *IL17REL* |
| 5 | 15 577 372 | 7.20 | *LRRC56* |
| 26 | 4 258 971 | 7.20 | *ANKS1A* |
| 1 | 131 183 892 | 7.20 | *GABRG3* |
| 10 | 2 692 283 | 7.20 | *SIN3A* |
| 1 | 16 119 147 | 7.19 | *HIST1H2B5L* |
| 1 | 16 119 147 | 7.19 | *GRAMD4* |
| 2 | 80 344 862 | 7.19 | *IKZF1* |
| 11 | 6 220 537 | 7.19 | *ADCY7* |

**Supplementary Table 7**

Forty-four candidate genes supported by overlapping F_ST_ and GWAS signals, with associated Gene Ontology (GO) enrichment terms.

| Gene Symbol | Description | Biological Process (GO) |
| --- | --- | --- |
| ASIC2 | acid sensing ion channel subunit 2 | GO:0003026 regulation of systemic arterial blood pressure by aortic arch baroreceptor feedback; GO:0003025 regulation of systemic arterial blood pressure by baroreceptor feedback; GO:0050915 sensory perception of sour taste |
| ATP5E | ATP synthase F1 subunit epsilon | GO:0042776 proton motive force-driven mitochondrial ATP synthesis; GO:0015986 proton motive force-driven ATP synthesis; GO:0006754 ATP biosynthetic process |
| CAMTA1 | calmodulin binding transcription activator 1 | GO:0070886 positive regulation of calcineurin-NFAT signaling cascade; GO:0106058 positive regulation of calcineurin-mediated signaling; GO:0070884 regulation of calcineurin-NFAT signaling cascade |
| CASS4 | Cas scaffold protein family member 4 | GO:0061098 positive regulation of protein tyrosine kinase activity; GO:0061097 regulation of protein tyrosine kinase activity; GO:1900026 positive regulation of substrate adhesion-dependent cell spreading |
| CDKL5 | cyclin dependent kinase like 5 | GO:0035022 positive regulation of Rac protein signal transduction; GO:0035020 regulation of Rac protein signal transduction; GO:0050775 positive regulation of dendrite morphogenesis |
| DAAM2 | dishevelled associated activator of morphogenesis 2 | GO:0090521 podocyte cell migration; GO:0048715 negative regulation of oligodendrocyte differentiation; GO:0021516 dorsal spinal cord development |
| DDX27 | DEAD-box helicase 27 | GO:0006364 rRNA processing; GO:0016072 rRNA metabolic process; GO:0042254 ribosome biogenesis |
| EDN3 | endothelin 3 | GO:0014826 vein smooth muscle contraction; GO:0003100 regulation of systemic arterial blood pressure by endothelin; GO:0010961 intracellular magnesium ion homeostasis |
| FAM19A5 | TAFA chemokine like family member 5 | GO:0061044 negative regulation of vascular wound healing; GO:0061043 regulation of vascular wound healing; GO:1904706 negative regulation of vascular associated smooth muscle cell proliferation |
| GABRG3 | gamma-aminobutyric acid type A receptor subunit gamma3 | GO:1904862 inhibitory synapse assembly; GO:0007214 gamma-aminobutyric acid signaling pathway; GO:0051932 synaptic transmission, GABAergic |
| GRM8 | glutamate metabotropic receptor 8 | GO:0007196 adenylate cyclase-inhibiting G protein-coupled glutamate receptor signaling pathway; GO:0007216 G protein-coupled glutamate receptor signaling pathway; GO:0007215 glutamate receptor signaling pathway |
| HERC1 | HECT and RLD domain containing E3 ubiquitin protein ligase family member 1 | GO:0021702 cerebellar Purkinje cell differentiation; GO:0021694 cerebellar Purkinje cell layer formation; GO:0021692 cerebellar Purkinje cell layer morphogenesis |
| HERC3 | HECT and RLD domain containing E3 ubiquitin protein ligase 3 | GO:0006511 ubiquitin-dependent protein catabolic process; GO:0019941 modification-dependent protein catabolic process; GO:0043632 modification-dependent macromolecule catabolic process |
| HSF5 | heat shock transcription factor 5 | GO:0007140 male meiotic nuclear division; GO:0140013 meiotic nuclear division; GO:1903046 meiotic cell cycle process |
| ITGB8 | integrin subunit beta 8 | GO:0061520 Langerhans cell differentiation; GO:0060022 hard palate development; GO:0043011 myeloid dendritic cell differentiation |
| KIAA1217 | KIAA1217 | GO:0048706 embryonic skeletal system development; GO:0001501 skeletal system development; GO:0043009 chordate embryonic development |
| KIF26B | kinesin family member 26B | GO:0072092 ureteric bud invasion; GO:0072093 metanephric renal vesicle formation; GO:0060677 ureteric bud elongation |
| LHFPL1 | LHFPL tetraspan subfamily member 1 |  |
| LOC101747896 | None | None |
| LOC112530078 | None | None |
| MACROD2 | mono-ADP ribosylhydrolase 2 | GO:0140291 peptidyl-glutamate ADP-deribosylation; GO:0051725 protein de-ADP-ribosylation; GO:0042278 purine nucleoside metabolic process |
| MC1R | melanocortin 1 receptor | GO:2000253 positive regulation of feeding behavior; GO:0009650 UV protection; GO:0070914 UV-damage excision repair |
| MTMR4 | myotubularin related protein 4 | GO:2001136 negative regulation of endocytic recycling; GO:2001135 regulation of endocytic recycling;GO:0061952 midbody abscission |
| NRG3 | neuregulin 3 | GO:0021840 directional guidance of interneurons involved in migration from the subpallium to the cortex; GO:0021842 chemorepulsion involved in interneuron migration from the subpallium to the cortex; GO:0060596 mammary placode formation |
| NRXN1 | neurexin 1 | GO:0090126 protein-containing complex assembly involved in synapse maturation; GO:1905518 regulation of presynaptic active zone assembly; GO:1905520 positive regulation of presynaptic active zone assembly |
| PDE4B | phosphodiesterase 4B | GO:1901898 negative regulation of relaxation of cardiac muscle; GO:0071878 negative regulation of adenylate cyclase-activating adrenergic receptor signaling pathway; GO:1901897 regulation of relaxation of cardiac muscle |
| PPP2R3B | protein phosphatase 2 regulatory subunit B''beta | GO:0006470 protein dephosphorylation; GO:0016311 dephosphorylation; GO:0051726 regulation of cell cycle |
| PRELID3B | PRELI domain containing 3B | GO:0120009 intermembrane lipid transfer; GO:0015914 phospholipid transport; GO:0015748 organophosphate ester transport |
| RNF43 | ring finger protein 43 | GO:0038018 Wnt receptor catabolic process; GO:0032801 receptor catabolic process; GO:0043112 receptor metabolic process |
| RTFDC1 | replication termination factor 2 | GO:0097752 regulation of DNA stability; GO:0072711 cellular response to hydroxyurea; GO:0072710 response to hydroxyurea |
| SDK1 | sidekick cell adhesion molecule 1 | GO:0048148 behavioral response to cocaine; GO:0010842 retina layer formation; GO:0042220 response to cocaine |
| SH3KBP1 | SH3 domain containing kinase binding protein 1 | GO:0050871 positive regulation of B cell activation; GO:0050864 regulation of B cell activation; GO:0008360 regulation of cell shape |
| SPIRE2 | spire type actin nucleation factor 2 | GO:0070649 formin-nucleated actin cable assembly; GO:0110009 formin-nucleated actin cable organization; GO:0040038 polar body extrusion after meiotic divisions |
| STAG3 | STAG3 cohesin complex component | GO:0034089 establishment of meiotic sister chromatid cohesion; GO:0034085 establishment of sister chromatid cohesion; GO:0051177 meiotic sister chromatid cohesion |
| STARD13 | StAR related lipid transfer domain containing 13 | GO:0097498 endothelial tube lumen extension; GO:0003159 morphogenesis of an endothelium; GO:0061154 endothelial tube morphogenesis |
| TCF25 | TCF25 ribosome quality control complex subunit | GO:0061945 regulation of protein K48-linked ubiquitination; GO:1902914 regulation of protein polyubiquitination; GO:0072344 rescue of stalled ribosome |
| TENM1 | teneurin transmembrane protein 1 | GO:0033138 positive regulation of peptidyl-serine phosphorylation; GO:0006359 regulation of transcription by RNA polymerase III; GO:0051491 positive regulation of filopodium assembly |
| TSNARE1 | t-SNARE domain containing 1 | GO:0048278 vesicle docking; GO:0140056 organelle localization by membrane tethering; GO:0022406 membrane docking |
| TUBB1 | tubulin beta 1 class VI | GO:0070327 thyroid hormone transport; GO:0030220 platelet formation; GO:0036344 platelet morphogenesis |
| TUBB3 | tubulin beta 3 class III | GO:1990791 dorsal root ganglion development; GO:0038007 netrin-activated signaling pathway; GO:0061548 ganglion development |
| TWISTNB | RNA polymerase I subunit F | GO:0006362 transcription elongation by RNA polymerase I; GO:0042790 nucleolar large rRNA transcription by RNA polymerase I; GO:0009303 rRNA transcription |
| ZDHHC20 | zDHHC palmitoyltransferase 20 | GO:0019062 virion attachment to host cell; GO:0044650 adhesion of symbiont to host cell; GO:0018230 peptidyl-L-cysteine S-palmitoylation |
| ZNF292 | zinc finger protein 292 | GO:0045944 positive regulation of transcription by RNA polymerase II; GO:0045893 positive regulation of DNA-templated transcription; GO:1902680 positive regulation of RNA biosynthetic process |
| ZNF831 | zinc finger protein 831 |  |

**Supplementary Table 8**

*HSF5* absolute relative allele frequency（>0.6）in carnation comb chicken against non-carnation comb chicken.

| Chr | Position | Variant type | Allele1 | Allele2 | Case_MAF | Control_MAF | RAFdif |
| --- | --- | --- | --- | --- | --- | --- | --- |
| 19 | 1130544 | intron_variant | C | G | 0.8482 | 0 | 0.8482 |
| 19 | 1132538 | intron_variant | G | C | 0.8661 | 0.074 | 0.7921 |
| 19 | 1129974 | intron_variant | C | A | 0.8214 | 0.0322 | 0.7892 |
| 19 | 1132414 | intron_variant | G | T | 0.8839 | 0.1867 | 0.6972 |
| 19 | 11132389 | intron_variant | C | G | 0.8839 | 0.1905 | 0.6934 |
| 19 | 1132451 | intron_variant | C | T | 0.875 | 0.1939 | 0.6811 |
| 19 | 1131061 | intron_variant | A | G | 0.6786 | 0.0029 | 0.6757 |
| 19 | 1134274 | upstream_gene_variant | C | T | 0.8661 | 0.2101 | 0.656 |
| 19 | 1134271 | upstream_gene_variant | G | C | 0.8661 | 0.2147 | 0.6514 |
| 19 | 1128611 | intron_variant | G | T | 0.6518 | 0.0029 | 0.6489 |
| 19 | 1134758 | upstream_gene_variant | G | T | 0.6518 | 0.0087 | 0.6431 |
| 19 | 1130314 | synonymous_variant | C | T | 0.9464 | 0.3088 | 0.6376 |
| 19 | 1137178 | upstream_gene_variant | C | T | 0.6429 | 0.0088 | 0.6341 |
| 19 | 1126993 | intron_variant | A | G | 0.6339 | 0 | 0.6339 |
| 19 | 1135971 | upstream_gene_variant | C | A | 0.6429 | 0.0116 | 0.6313 |
| 19 | 1131994 | intron_variant | A | G | 0.6964 | 0.0702 | 0.6262 |
| 19 | 1137250 | upstream_gene_variant | C | T | 0.6339 | 0.0087 | 0.6252 |
| 19 | 1127833 | intron_variant | G | A | 0.6161 | 0 | 0.6161 |
| 19 | 1127844 | intron_variant | G | A | 0.6071 | 0 | 0.6071 |

^1^ Chr = chromosome;

^2^ MAF: Frequency of mutant alleles

^3^ Case_MAF: The carnation comb chicken Allele1 frequency; Control_MAF: The non-carnation comb chicken Allele1 frequency;

^4^ RAFdif: relative allele frequencies; RAFdif =Case_MAF - Control_MAF;

**Supplementary Figure 1.** Principal component analysis (PCA) of the same individuals, showing the first six principal components (PC1 to PC6).

(a) scatter plot of PC1 and PC2; (b) scatter plot of PC2 and PC3; (c) scatter plot of PC3 and PC4; (d) scatter plot of PC4 and PC5; (e) scatter plot of PC5 and PC6.

**Supplementary Figure 2.** Cross-validation (CV) error for K = 1–11 in the ADMIXTURE-based population structure analysis.

**Supplementary Figure 3**. Distributions of individual-level genetic diversity metrics across the ten chicken breeds.

(a) inbreeding coefficient (*F*), (b) mean intra-population genetic distance (DST), and (c) total length of runs of homozygosity (ROH, in kb) per individual across the ten studied breeds. The horizontal line within each box represents the median, the box bounds represent the interquartile range (IQR), and whiskers extend to 1.5 times the IQR. Individual dots beyond the whiskers denote outliers. Distinct lowercase letters (a-g) positioned above the boxplots indicate statistically significant differences between breeds based on Tukey’s honestly significant difference (HSD) post-hoc test (α = 0.05) following a one-way analysis of variance (ANOVA).

**Supplementary Figure 4.** Significantly enriched Gene Ontology (GO) terms for candidate genes supported by overlapping F_ST_ and GWAS signals.

**Supplementary Figure 5.** Four comb phenotypes in chickens.

(A) Single-combed wild-type male, (B) Rose-combed male, (C) Pea-combed male, and (D) Walnut-combed male. Note: These images are reproduced from an open-access study (Imsland et al., 2012) under the Creative Commons Attribution License (CC BY 4.0). Original photography credits belong to Freyja Imsland (panels A–C) and David Gourichon (panel D).

**Supplementary References**

Imsland F, Feng C, Boije H, et al. The Rose-comb mutation in chickens constitutes a structural rearrangement causing both altered comb morphology and defective sperm motility[J]. PLoS genetics, 2012, 8(6): e1002775.
